# Ameliorative patterns of grey matter in patients with first-episode and treatment-naïve schizophrenia

**DOI:** 10.1017/S0033291722000058

**Published:** 2023-06

**Authors:** Mingli Li, Wei Deng, Yinfei Li, Liansheng Zhao, Xiaohong Ma, Hua Yu, Xiaojing Li, Yajing Meng, Qiang Wang, Xiangdong Du, Pak Chung Sham, Lena Palaniyappan, Tao Li

**Affiliations:** 1 Mental Health Center and Psychiatric Laboratory, State Key Laboratory of Biotherapy, West China Hospital of Sichuan University, Chengdu, China; 2 Affiliated Mental Health Center & Hangzhou Seventh People's Hospital, Zhejiang University School of Medicine, Hangzhou, Zhejiang, China; 3 Suzhou Psychiatry Hospital, Affiliated Guangji Hospital of Soochow University, Suzhou, 215137, Jiangsu, China; 4 Centre for Genomic Sciences and State Key Laboratory in Cognitive and Brain Sciences, LKS Faculty of Medicine, The University of Hong Kong, Pokfulam, Hong Kong, China; 5 Robarts Research Institute & The Brain and Mind Institute, University of Western Ontario, London, Ontario, Canada; 6 Department of Psychiatry, University of Western Ontario, London, Ontario, Canada; 7 Lawson Health Research Institute, London, Ontario, Canada

**Keywords:** psychosis, morphometry, compensatory, neurocognition, early intervention

## Abstract

**Background:**

Grey matter (GM) reduction is a consistent observation in established late stages of schizophrenia, but patients in the untreated early stages of illness display an increase as well as a decrease in GM distribution relative to healthy controls (HC). The relative excess of GM may indicate putative compensatory responses, though to date its relevance is unclear.

**Methods:**

343 first-episode treatment-naïve patients with schizophrenia (FES) and 342 HC were recruited. Multivariate source-based morphometry was performed to identify covarying ‘networks' of grey matter concentration (GMC). Neurocognitive scores using the Cambridge Neuropsychological Test Automated Battery (CANTAB) and symptom burden using the Positive and Negative Symptoms Scale (PANSS) were obtained. Bivariate linear relationships between GMC and cognition/symptoms were studied.

**Results:**

Compared to healthy subjects, FES had prominently lower GMC in two components; the first consists of the anterior insula, inferior frontal gyrus, anterior cingulate and the second component with the superior temporal gyrus, precuneus, inferior/superior parietal lobule, cuneus, and lingual gyrus. Higher GMC was seen in adjacent areas of the middle and superior temporal gyrus, middle frontal gyrus, inferior parietal cortex and putamen. Greater GMC of this component was associated with lower duration of untreated psychosis, less severe positive symptoms and better performance on cognitive tests.

**Conclusions:**

In untreated stages of schizophrenia, both a distributed lower and higher GMC is observable. While the higher GMC is relatively modest, it occurs across frontoparietal, temporal and subcortical regions in association with reduced illness burden suggesting a compensatory role for higher GMC in the early stages of schizophrenia.

## Introduction

Progressive loss of grey matter (GM) tissue is one of the presumed pathophysiological processes targeted by the paradigm of ‘early intervention’ in psychosis (Lieberman, Small, & Girgis, 2019; Palaniyappan, 2021). Several longitudinal observations report a progressive loss of GM in patients with schizophrenia, occurring at a rate greater than expected from healthy individuals (Vita, De Peri, Deste, & Sacchetti, 2012). This lower GM appears to be spatially constrained to key cortical regions (frontoinsular and temporal cortex), and temporally limited to the period immediately after the first episode (Palaniyappan, 2017). GM reduction also appears to be more pronounced in patients with severe cognitive impairment at the time of the first presentation, though its relationship with symptom burden at presentation is still unclear (Takahashi & Suzuki, 2018).

Recently emerging evidence indicates that a subtle but progressive increase in the GM may occur alongside a progressive loss in patients with schizophrenia. This gain appears to be spatially more diffuse and not identifiable by conventional mass univariate analyses. Nevertheless, the relative increase becomes apparent in covariance analyses (Guo, Palaniyappan, Liddle, & Feng, 2016; Kasparek et al., 2010; Palaniyappan et al., 2015). Lv et al. recently used normative modelling and reported that while 79% of patients with schizophrenia show infra-normal deviations in brain structure (i.e. <5th percentile value of age- and sex-matched healthy individuals), 46% show supra-normal deviations (i.e. >95th percentile value) for at least one brain region (Lv et al., 2021). Areas of higher GM were less regionally concentrated than lower GM reduction. Mancuso and colleagues used a meta-analytical network mapping approach and concluded that whenever GM reduction occurs in one brain region, concurrent GM increase occurs in other brain regions in schizophrenia (Mancuso et al., 2020). In addition, meta-analytic evidence of cross-sectional studies indicates that while some regional GM changes persist or expand in chronic schizophrenia, others ameliorate, ‘closing the gap’ with healthy controls (HC) (Liloia et al., 2021). Taken together, these findings support a process of reorganisation characterised by concurrent GM loss as well as gain in schizophrenia. At present, it is unclear if the GM increase occurs even before the onset of treatment (thus an inherent process independent of medication exposure), and if such increases are indeed associated with a favourable clinical profile [thus a compensatory process (Palaniyappan, 2019)].

In this work, we investigate the relative increases and decreases in GM in the largest ever-reported sample of first-episode neuroleptic-naïve patients with schizophreniform psychosis and schizophrenia (NN-FES, *n* = 343) and matched healthy subjects (*n* = 342). As patients with a more severe illness often receive a higher antipsychotic dose, which is independently associated with GM reduction (Voineskos et al., 2020) as well as an increase (Stip et al., 2009), the recruitment of a challenging sample of fully NN-FES is crucial to study the possibility of a compensatory amelioration. Besides recruiting the sample of NN-FES, we also employ a covariance analysis that utilises a multivariate approach as opposed to the mass univariate approaches of voxel-based morphometry (VBM). The mass univariate approach of VBM search fails to consider the relationship among brain regions in structural changes introduced by maturational or neuroplastic processes, and the intrinsic connectivity (‘common fate’). Source-based morphometry (SBM) is a multivariate extension of VBM with independent component analysis identifying naturally grouping, maximally independent sources; the identified ‘source networks’ provide information about localisation of GM changes and their variation among individuals (Kasparek et al., 2010; Xu, Groth, Pearlson, Schretlen, & Calhoun, 2009). This approach also increases the sensitivity of detecting anatomical changes by compartmentalising noise and scanner effects, and allows distributed brain regions sharing ‘common fate’ to be studied as single units (Gupta et al., 2015). As a result, the ability to detect a concurrent GM increase that accompanies a GM decrease is improved when employing SBM.

In the present study, we hypothesise that a relative increase in GM, compared to healthy individuals, occurs alongside more widespread GM reduction in the early stages of schizophrenia. We expect this relative increase to occur in those with more favourable cognitive and symptom profiles even before the onset of treatment. We also investigate the effects of the duration of untreated illness, symptom burden and cognitive function across multiple domains to address the question of compensatory cortical reorganisation characterised by concurrent GM deficits and excesses in schizophrenia.

## Material and methods

### Subjects

343 first-episode neuroleptic-naïve patients with schizophreniform psychosis and schizophrenia (NN-FES) (age from 16 to 45 years) from the Mental Health Center in West China Hospital and 342 HC (age from 16 to 45 years) were enrolled in the present study. Patients were interviewed and assessed using the Structured Clinical Interview for the DSM-IV (Diagnostic and Statistical Manual of Mental Disorders, fourth edition) (SCID-I/P) (First, Spitzer, & Williams, 1997), and fulfilled diagnostic criteria for schizophrenia or schizophreniform psychosis in the DSM-IV. Subjects with evidence of organic brain disorders, alcohol or operationally defined ‘drug abuse’, or any other severe physical illness such as brain tumour or epilepsy, coronary heart disease, hyperthyroidism, serious kidney or liver disease and pulmonary infection were excluded. The ‘drug abuse’ definition was based on the drug list in SCID-I including sedative, cannabis, stimulant, opioid, cocaine, hallucinogen, phencyclidine and others (e.g. steroid medicines, diet pills).

342 HC were recruited from the local area by poster advertisements. All controls were screened for the lifetime absence of psychiatric illnesses by using the SCID non-patient version (SCID-I/NP) (First, SR, & Williams, 1996). In addition, control subjects were interviewed to ascertain that there was no psychiatric illness among their first-degree relatives.

All participants were Han Chinese and right-handed. The handedness of participants was assessed with Annett Handedness Scale (Annett, 1970). This study was carried out in accordance with the Declaration of Helsinki and was approved by the Institutional Review Board of West China Hospital, Sichuan University (2005NO.34 & 2013NO.89). After a complete description of the study to the subjects, written informed consent was obtained from the HC and patients and their guardians when appropriate.

The severity of symptoms was evaluated using the 30-item Positive and Negative Symptoms Scale (PANSS) (Kay, Flszbein, & Opfer, 1987). PANSS scores were available for 295 of 343 NN-FES subjects. Neurocognition was assessed using the Cambridge Neuropsychological Test Automated Battery (CANTAB) including six computerised tasks [Rapid Visual Information Processing (RVP), Pattern Recognition Memory (PRM), Delayed Matching to Sample (DMS), Spatial Working Memory (SWM), Intra-Extra Dimensional Set Shift (IED), Stockings of Cambridge (SOC)] from the CANTAB-eclipse running on a personal computer with a high-resolution touchscreen. For more detailed descriptions of these tests see the CANTAB^®^ website (www.cambridgecognition.com). 210 of 343 NN-FES and 253 of 342 HC had CANTAB data available. A total of 214 NN-FES and 292 HC were assessed using the short version of the seven-subtest (information, arithmetic, digital symbol, digital span test, block design, picture completion, and similarities) Chinese Revised Version of the Wechsler Adult Intelligence Scale (WAIS-RC) (Gong, 1992). CANTAB and PANSS were administered within two days of the scan in all cases.

### Magnetic resonance imaging (MRI) data acquisition

Patients were scanned after written informed consent was obtained, but before treatment initiation; in all cases, this was on the same day of initial assessment, along with other laboratory assessments required for routine clinical care. Patients were not included in the study if any exposure to antipsychotics took place before the scanning. 206 patients and 170 HC were scanned on a Signa 3.0-T MR scanner [General Electric (GE), Medical Systems, USA] using an eight-channel phased-array head coil in the Department of Radiology at West China Hospital. The scan parameters are listed in the Supplementary Methods. 137 patients and 172 HC were scanned on a 3.0 T MR scanner (Philips Achieva, Amsterdam, the Netherlands) using an eight-channel phased-array head coil in the department of Radiology at West China Hospital Tibet People's Government in Chengdu Office Branch. The data from the 2 scanners (datasets 1 and 2 respectively) were combined for aggregated analysis; see online supplementary materials for scanner-related variations.

### T1 image preprocessing and source-based morphometry (SBM)

To improve the registration of the MRI images, T1 Images were processed using Diffeomorphic Anatomical Registration Through Exponentiated Lie algebra (DARTEL) toolbox in Statistical Parametric Mapping (SPM) 8, and followed the standard approach of Ashburner (2007). The details of VBM preprocessing is shown in the Supplementary Methods. Finally, these unmodulated grey-matter images were smoothed with an isotropic Gaussian kernel of 6-mm full-width, half-maximum to be used in the next step analysis.

The SBM module of the GIFT Toolbox (http://mialab.mrn.org/software/gift/) was used to perform the independent component analysis decompositions on the aggregated dataset consisting of 343 NN-FES and 342 HC from 2 scanners (Xu et al., 2009). The number of components was set to 30 as previous studies suggested (Gupta et al., 2015; Xu et al., 2009). We employed a bootstrapping approach [ICASSO: (http://research.ics.aalto.fi/ica/icasso/) (Himberg, Hyvarinen, & Esposito, 2004)] with 20 iterations to resample the available data while retaining the structure within the data; this simulated a random sampling process, providing estimates of the statistical reliability of components, and allowing the unstable components to be discarded. The subjects-by-voxels matrix was decomposed into a mixing matrix (subjects-by-components) representing loading parameters that quantify the contribution of each subject to the group for a given component and source matrix (components-by-voxels) representing the spatially independent ‘sub-systems’ defined on the basis of morphometric covariance within the group as in our previous study (Palaniyappan et al., 2015). Artefact components were identified visually and not included in subsequent analyses. To confirm the robustness of these findings across the scanners, we also performed separate SBM decompositions for the 2 datasets.

To visualise the spatial components, the source matrix was recomposed to statistical maps in 3-dimensional MNI space with coefficients expressed in standard deviation units (z-maps). These source maps were scaled to unit standard deviation (SBM Z map) and thresholded at a value of |*Z*| > 3:0. The anatomical descriptions of these maps were obtained using Talairach coordinates utility in the GIFT toolbox.

The GM intensity in each voxel in each subject (after removal of the group mean) is the sum of 30 product values obtained from multiplying the voxel loading for each component with its coefficient for that subject. The sign of the loading coefficients of a component in a subject does not directly provide the direction of change in absolute GM intensity in a region (Gupta et al., 2015). As a result, interpretation of the loading coefficients of the spatial ICA GM maps needs some care. A larger loading coefficient for an individual or group indicates that the spatial pattern is more strongly weighted in the data for that individual or group, but the interpretation of the loading coefficient difference depends upon the spatial image of the component. If a spatial component is predominantly positive, with the loading coefficients greater in HC than NN-FES, we infer that grey matter concentration (GMC) is greater in HC for the spatial component under consideration. If a spatial component is predominantly negative, with the loading coefficients being greater in HC than NN-FES, GMC is lower in HC compared to NN-FES for the spatial component under consideration.

We also conducted a VBM analysis comparing the 2 groups (details in the online Supplement).

### Statistical analysis

#### Demographic and clinical characteristics

Independent two-sample *t* tests (continuous variables) or χ^2^ tests (categorical variables) were used to evaluate differences in demographic and clinical characteristics between NN-FES and HC. All statistical tests were carried out using SPSS, Version 18.0. (IBM, Armonk, New York).

#### The differences in loading coefficients between NN-FES and HC participants

A multivariate analysis of covariance (MANCOVA) model was used with SBM loading coefficients as dependent variables, diagnosis as a factor, and scanner, age, gender, years of education and intracranial volume as covariates in SPSS 18.0. A threshold of *p* < 0.05 after correcting for multiple testing using the false discovery rate (FDR) method was used to infer statistical significance for group comparisons. SBM analysis was repeated on the two scanner-specific datasets separately to confirm the results from the combined analysis (See online Supplement section 4).

#### Principal component analysis for neurocognition from CANTAB

Principal component analysis (PCA) was used for dimensionality reduction across the 16 scores from 6 CANTAB subtests. The latent variables from the PCA were assumed to reflect underlying cognitive dimensions. For this PCA, a parallel analysis (PA) helped determine the most appropriate number of components to be retained. As the components were expected to be correlated, we employed a varimax rotation (Further details in the Supplement).

#### Correlates of clinical and neurocognitive variables with SBM loading coefficients

To exclude the effects of scanner, age, sex, and years of education on loading parameters of components, multiple regression analyses were performed on each SBM component as dependent variables, and scanner, age, gender, educational years and intracranial volume as independent variables, in the HC and NN-FES separately. Standard residual scores of each component from these regressions were then substituted for the raw values in the correlation analyses with clinical and neurocognitive variables.

As the duration of the untreated psychosis (DUP) showed a skewed distribution, Spearman's test was used to assess the relationship between DUP and the SBM components showing group difference. Pearson's test was used to evaluate the relationship between the SBM components and positive, negative, and disorganisation factor scores from PANSS (van der Gaag et al., 2006) as well as age in the patient group for the identified SBM components (We report multiple regression analyses at a whole-brain level in the supplement). Additionally, when examining the correlation between SBM components and 4 cognitive factors from PCA of CANTAB scores, we used partial correlation adjusting for full-scale IQ. We also examined the relationship between components showing lower and higher GM in patients using Pearson's test.

Correlations were deemed statistically significant based on an FDR-corrected *p* < 0.05 threshold.

## Results

### Demographic and clinical characteristics

Demographic, clinical, and neurocognitive features of the sample are presented in Table 1. The two groups were well matched for age and gender. The NN-FES had lower number of years of education and intelligence quotient (IQ) scores than HC, as expected (Also see online Supplementary Tables S1 and S2). Patients scanned with the GE system were more symptomatic than the other group scanned with the Phillips scanner, but there were no differences in age, DUP or neurocognition (see online Supplementary materials).

**Table 1.** Demographics and clinical information of aggregate data

	NN-FES (*n* = 343)	Control (*n* = 342)	df	*T*/χ^2^ value	*p* value
Age (years)	23.76 (7.10)	24.33 (5.68)	683	−1.16	0.25
Years of education	11.96 (2.99)	14.74 (2.30)	683	−12.16	0.00
Gender (M/F)	170/173	147/195	1	2.98	0.09
Age of onset	22.83 (7.14)				
DUP (months)	4 (1; 15)^a^				
Positive	26.30 (6.20)				
Negative	23.60 (9.81)				
Disorganisation	32.17 (7.66)				
IQ	99.65 (13.63)	118.12 (11.56)	504	−16.45	0.00

NN-FES, first-episode neuroleptic-naïve patients with schizophreniform psychosis and schizophrenia; DUP, duration of untreated psychosis; M/F, male/female; IQ, intelligence quotient.

a Median (quarter; three quarters).

#### SBM results

**Fig. 1.**
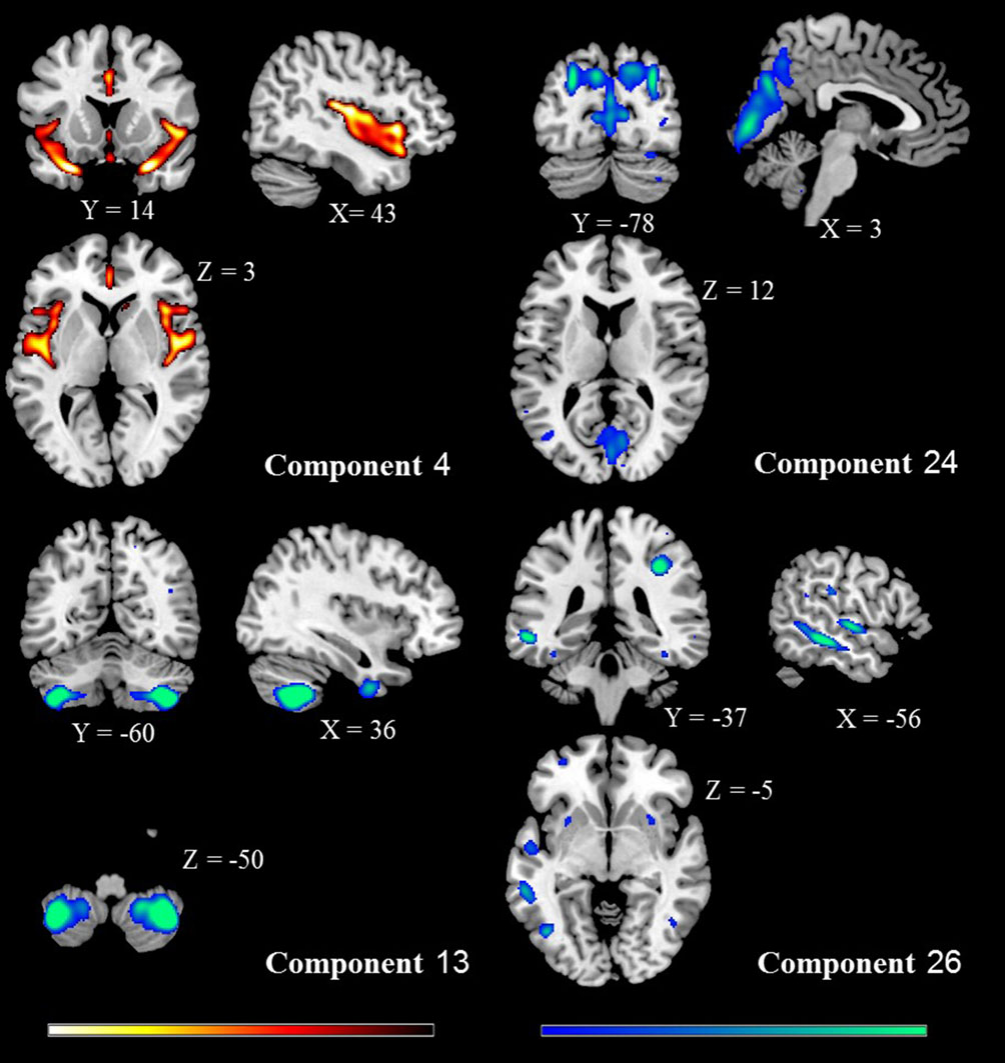
Spatial maps of the four components showing (NN-FES *v*. HC) group effect. All are thresholded at |*z*| > 3 and superimposed onto a standard brain template provided by MRICron. The colour bar indicates the colour mapping for the normalised component weights. HC > NN-FES: component 4, 24; NN-FES > HC: component 13, 26. SBM, source-based morphometry; HC, healthy control; NN-FES, first-episode neuroleptic-naïve patients with schizophreniform psychosis and schizophrenia.

**Fig. 2.**
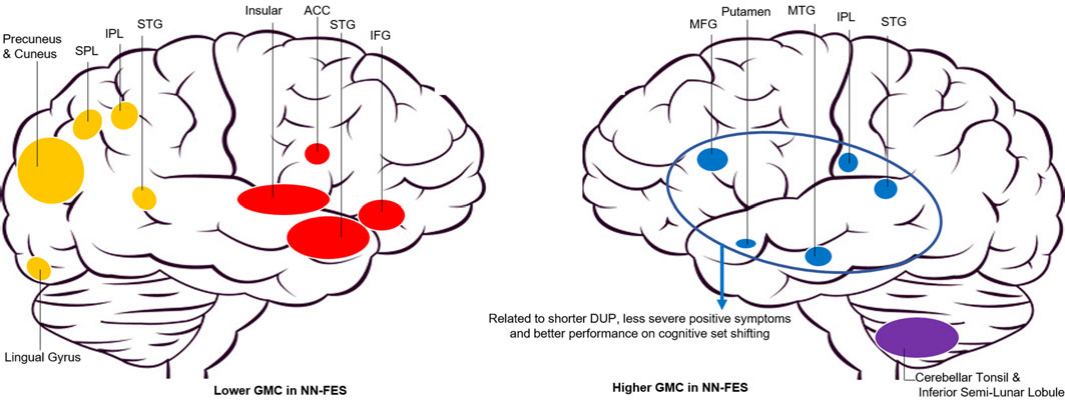
Patterns of grey matter alterations in first-episode neuroleptic-naïve patients with schizophreniform psychosis and schizophrenia. Abbreviations: GMC, grey matter concentration; NN-FES, first-episode neuroleptic-naïve patients with schizophreniform psychosis and schizophrenia; SPL, Superior Parietal Lobule; IPL, Inferior Parietal Lobule; STG, Superior Temporal Gyrus; ACC, Anterior Cingulate Cortex; IFG, Inferior Frontal Gyrus; MTG, Middle Temporal Gyrus. Component 4 including insular, ACC, STG and IFG regions is shown in red; Component 24 including precuneus and cuneus, lingual gyrus, SPL, IPL and STG is shown in yellow; Component 26 including MFG, MTG, IPL, STG and putamen is shown in blue; Component 13 including cerebellar Tonsil and inferior Semi-Lunar Lobule is shown in purple.

The tissue maps of GMC were decomposed into 30 SBM components. Eight components were suggestive of obvious artefacts, i.e. having spatial patterns primarily around the edges of the brain, in white matter regions or CSF and these were discarded (online Supplementary Fig. S7). From the remaining components, we observed four spatial patterns showing a significant effect of diagnosis. These four components and their spatial patterns are depicted in Fig. 1 and Table 2 with brain regions having volumes greater than 1 cm^3^, labelled in the order of decreasing effect sizes. Components 4 and 24 included areas where GMC values were greater in HC than NN-FES. Component 4 included insula, inferior frontal gyrus, anterior cingulate and superior temporal gyrus. Component 24 included precuneus, inferior/superior parietal lobule, cuneus, and lingual gyrus. In contrast, component 13 (including cerebellar tonsil and inferior semi-lunar lobule) and component 26 (including middle and superior temporal gyrus, middle frontal gyrus and putamen) had areas with higher GMC in NN-FES than HC (Fig. 2). The brain labels in Table 2 were obtained from the Talairach Daemon (http://www.talairach.org/daemon.html) based on the transformed locations of the largest clusters in the component maps. All four components had similar diagnostic differences when scanner-specific subsets of data were analysed, but a component with spatial similarity to component 13 was not recovered independently from the Philips scanner (see online Supplementary materials for scanner effects).

**Table 2.** Loading directionality and brain labels for the spatial components showing NN-FES /HC difference

Component number	Loadings directionality	Brain region label	Brodmann area	L/R Volume (cm^3^)	Talairach (*x*, *y*, *z*)	MNI (*x*, *y*, *z*)	*F* value	*p* value (FDR Corrected)	GMC Effect size^a^ (partial eta squared)
4	HC > NN-FES	Superior Temporal Gyrus	13, 21, 22, 38, 41, 42	6.5/4.9	(−25, 10, −24)/(27, 13, −23)	(−25.5, 12, −28.5)/(27, 15, −27)	27.33	<0.001	0.039
		Insula	13, 22, 40, 41, 47	5.5/5.5	(−43, −16, 10)/(45, −13, 10)	(−43.5, −16.5, 10.5)/(45, −13.5, 10.5)			
		Inferior Frontal Gyrus	9, 13, 44, 45, 47	3.7/4.2	(−37, 17, −18)/(31, 16, −22)	(−37.5, 18, −21)/(31.5, 18, −25.5)			
		Anterior Cingulate	24, 25, 32, 33	1.6/1.6	(−1, 26, 25)/(1, 42, −3)	(−15, 25.5, 28.5)/(15, 43.5, −15)			
24	HC > NN-FES	Precuneus	7, 19, 23, 31	6.8/7.3	(−9, −71, 34)/(30, −73, 35)	(−9, −75, 33)/(30, −76.5, 34.5)	12.30	0.004	0.018
		Cuneus	7, 17, 18, 19, 23, 30, 31	4.6/4.2	(−10, −70, 31)/(12, −67, 32)	(−10.5, −73.5, 30)/(12, −70.5, 31.5)			
		Inferior Parietal Lobule	7, 39, 40	1.6/1.6	(−36, −43, 45)/(36, −45, 38)	(−36, −46.5, 46.5)/(36, −48, 39)			
		Lingual Gyrus	17, 18	1.2/1.5	(0, −86, 3)/(1, −83, 4)	(0, −88.5, −15)/(15, −85.5, 0)			
		Superior Parietal Lobule	7, 40	1.2/1.2	(−24, −66, 43)/(30, −68, 43)	(−24, −70.5, 43.5)/(30, −72, 43.5)			
		Superior Temporal Gyrus	22, 39, 41, 42	1.1/0.0	(−43, −55, 29)/(45, −60, 21)	(−43.5, −58.5, 28.5)/(45, −63, 19.5)			
13	NN-FES > HC	Cerebellar Tonsil	NA	5.2/6.2	(−37, −54, −38)/(36, −54, −38)	(−37.5, −54, −48)/(36, −54, −48)	12.70	0.004	0.018
		Inferior Semi-Lunar Lobule	*	1.2/1.5	(−34, −63, −36)/(39, −62, −37)	(−34.5, −63, −46.5)/(39, −61.5, −48)			
26	NN-FES > HC	Middle Temporal Gyrus	20, 21, 22, 37, 39	2.6/1.2	(−56, −32, −6)/(59, −32, −7)	(−57, −33, −9)/(60, −33, −10.5)	7.91	0.028	0.012
		Middle Frontal Gyrus	20	2.3/2.8	(−36, 23, 21)/(34, −34, 40)	(−36, 22.5, 24)/(34.5, −37.5, 42)			
		Superior Temporal Gyrus	13, 21, 22, 38, 39	1.9/0.6	(−45, −55, 29)/(45, −46, 20)	(−45, −58.5, 28.5)/(45, −48, 19.5)			
		Middle Frontal Gyrus	6, 8, 9, 10, 46	1.0/1.1	(−36, 8, 38)/(34, 25, 28)	(−36, 6, 42)/(34.5, 24, 31.5)			
		Inferior Parietal Lobule	40	0.3/1.8	(−53, −25, 25)/(36, −34,40)	(−54, −27,25.5)/(36, −37.5, 42)			
		Putamen	NA	0.1/0.4	(−28, 10, −6)/(30, 8, −8)	(−28.5, 10.5, −6)/(30, 9, −9)			

*Note*: HC, healthy control; NN-FES, first-episode neuroleptic-naïve patients with schizophreniform psychosis and schizophrenia; MNI, Montreal neurological institute; FDR, false discovery rate. >

a Effect sizes were computed after extracting mean GMC from the spatial components from each subject; these were not calculated from loading coefficients.

#### PCA of CANTAB scores

Four distinctly interpretable components explaining 77.15% of the total variance emerged from 16 variables in 6 CANTAB subtests with the PCA. PC1 included five CANTAB subtest variables (RVP, SWM, DMS, PRM and SOC) representing sustained attention, memory and planning ability (Higher score is better for the neurocognitive ability of PC1) (factor loadings 0.53–0.90); PC2 included the CANTAB subtest variable IED reflecting cognitive set-shifting (lower score of PC 2, the better cognitive set-shifting ability) (factor loadings 0.95–0.97); PC3 was comprised of RVP Probability of false alarm (RVP-PFA) and RVP B″ (measure the tendency to respond regardless of whether the target sequence is present) (factor loadings 0.93) (higher is better); PC4 was comprised of DMS per cent correct, DMS per cent correct in *n* ms delay and Pattern Recognition Memory per cent correct in immediate test (factor loadings 0.48–0.82) (Higher score, better immediate memory). (See online Supplementary Table S6 and Fig. S3). NN-FES showed impaired neurocognition in all four components when compared to HC (Table 3).

**Table 3.** Differences in neurocognitive components between NN-FES and HC

CANTAB	NN-FES (*n* = 210)	HC (*n* = 253)	df	*T* value	*p* value
Component 1	−0.63 (0.90)	0.52 (0.74)	461	15.07	<0.00
Component 2	−0.26 (1.31)	0.22 (0.56)	461	5.22	<0.00
Component 3	−0.11 (1.37)	−0.09 (0.50)	461	2.07	0.04
Component 4	−0.22 (1.19)	0.18 (0.75)	461	4.43	<0.00

*Note*: NN-FES, first-episode neuroleptic-naïve patients with schizophreniform psychosis and schizophrenia; HC, health control; CANTAB, Neurocognitive scores using the Cambridge Neuropsychological Test Automated Battery. Cognitive scores contributing to each of the 4 components were numerically higher in healthy subjects than in patients. For ease of interpretation, the factor loading signs are retained for components 1, 3 and 4, but reversed for component 2, with lower negative or higher positive loading representing better cognitive ability. For the *t* test, absolute values of the *t* statistic are presented.

#### Clinical and cognitive correlates of GMC

Component 26 (with contributions from middle frontal, superior temporal gyrus and putamen) significantly related to the duration of psychosis (*r* = 0.152, FDR-corrected for clinical features *p* = 0.04), positive symptoms (*r* = 0.178, FDR-corrected for clinical features *p* = 0.04) and to neurocognitive factor 2 (adjusted for IQ) (*r* = 0.220, FDR-corrected for cognitive components *p* = 0.056) (Table 4). This indicated that shorter DUP, less severe positive symptoms, and better performance on IED are associated with greater GMC in the regions included in component 26 – middle frontal, superior temporal gyrus and putamen. In addition, variance in age is significantly related to the variance in loading coefficients of component 26 (*r* = 0.218, FDR-corrected *p* = 9.2 × 10^−5^) and component 4 (*r* = −0.246, FDR-corrected *p* = 1.6 × 10^−5^) among patients. The observed direction of this relationship indicated that younger patients had higher GMC for both components, but the *R*^2^ of these models were small (4.7–6.04%) (online Supplementary Fig. S4). No other significant correlation was found between GM spatial components, clinical or neurocognitive variables.

**Table 4.** Correlates of clinical and neurocognitive variables with SBM loadings in aggregate data

	C4	C24	C13	C26
DUP	0.030 (0.611)	−0.035 (0.551)	0.027 (0.643)	**0.152 (0.010)**
Positive	−0.018 (0.755)	−0.115 (0.049)	−0.020 (0.728)	**0.178 (0.002)**
Negative	−0.066 (0.260)	−0.019 (0.749)	−0.018 (0.753)	−0.079 (0.179)
Disorganisation	0.041 (0.482)	−0.077 (0.190)	0.017 (0.773)	0.028 (0.634)
CANTAB				
Component 1	−0.098 (0.236)	0.137 (0.095)	−0.093 (0.261)	0.187 (0.022)
Component 2	−0.102 (0.214)	−0.033 (0.691)	0.140 (0.090)	**0.220 (0.007)**
Component 3	−0.060 (0.464)	−0.009 (0.910)	0.074 (0.370)	−0.079 (0.338)
Component 4	0.073 (0.375)	0.084 (0.310)	−0.118 (0.150)	0.060 (0.466)

*Note*: *R* (uncorrected *p* value); False Discovery Rate-corrected for clinical features (p=0.04) or cognitive components (p=0.056) shown in bold; DUP, duration of untreated psychosis. Spearman correlation analysis for DUP; Bivariate correlation analysis for PANSS; Partial correlation analysis for neurocognition factors with IQ as covariate.

#### Correlation between components showing lower and higher GMC in patients

In the patient group, the loading on component 4 was significantly related to component 24 (*r* = −0.441, FDR-corrected *p* < 0.0001) and component 13 (*r* = 0.200, FDR-corrected *p* < 0.0001) and component 26 (*r* = −0.219, FDR-corrected *p* = 0.0001). The loading on component 24 was significantly related to component 13 (*r* = −0.208, FDR-corrected *p* < 0.0002). As component 4 was a predominantly positively loading spatial component while components 24, 13 and 26 were predominantly negative components, these correlations indicated that patients with lower GMC in one component (4 or 24) are likely to also have higher GMC in either component 13 (with lower GMC in component 4 or 24) or component 26 (with lower GMC in either component 4). No significant correlation was found between component 24 and component 26 as well as between component 13 and component 26 (online Supplementary Table S7), indicating that patients did not share the distribution of relative higher in GMC; while some patients had predominant higher GMC of component 13, others had a predominant higher GMC in component 26.

### VBM results

Compared with HC, patients with NN-FES had lower GMC in the bilateral cingulate gyrus, insular, right superior temporal gyrus, medial orbital frontal, rectus, caudate, precentral gyrus and lingual gyrus at FWE-corrected *p* < 0.05 and an extent threshold of 50 voxels, with no significant results for higher GMC in NN-FES group at this statistical threshold (online Supplementary Fig. S5*a*). However, when we used a more liberal threshold, higher GMC in the NN-FES group were found in caudate, cerebellum and a temporal pole at *p* < 0.001 at voxel level with FWE-corrected *p* < 0.05 at the cluster level, overlapping, and thus validating the interpreted direction of greater GMC in patients from the SBM analysis (online Supplementary Fig. S5*b*).

## Discussion

Using multivariate SBM in a sample of treatment-naïve first-episode patients with schizophrenia, we report three major findings. Firstly, we found both a prominently lower grey matter concentration in frontoinsular, temporal and parietal regions, and a higher GMC in a distributed cortical/subcortical component (middle and superior temporal gyrus, middle frontal gyrus and putamen), and cerebellum (tonsil and inferior semi-lunar lobule). Second, the higher GM in the cortico/subcortical component was seen in patients with shorter DUP and was associated with less severe positive symptoms and better performance on set-shifting ability, suggesting an adaptive advantage for patients with relative GM increase in these regions. Third, there was a significant relationship between the spatial components showing higher and lower GMC, supporting a coordinated reorganisation process. Taken together, these findings delineate a distributed pattern of GM changes in schizophrenia before any antipsychotic exposure.

When undertaking a VBM analysis, the subtle effect of higher GMC in the patient group was not detectable at FWE-corrected *p* < 0.05 for voxel level, but this became obvious at a lower threshold at *p* < 0.001 uncorrected at voxel level (online Supplementary Fig. S5). This supports our notion that in order to detect GM increases in schizophrenia, mass univariate approaches that depend on spatially contiguous clusters are less sensitive than approaches like SBM that leverage more distributed changes. Though the diagnostic effect of the cerebellar component did not independently replicate in the smaller of the 2 datasets, our observation of higher GM in NN-FES involving the cerebellum, middle and superior temporal gyrus, middle frontal gyrus, inferior parietal lobule and putamen is consistent with several studies with large sample size (>100 subjects) (Ren et al., 2013; Xiao et al., 2015) and the only previous study to use SBM in first-episode schizophrenia (Kasparek et al., 2010). In particular, increased thickness in overlapping cortical regions was reported in 128 patients with NN-FES (Xiao et al., 2015) and in brainstem and cerebellum in chronic and medicated patients with schizophrenia in a mega-analysis of 784 patients (Gupta et al., 2015). A recent meta-analysis has established a relative increase in GM in patients with chronic compared to recent-onset schizophrenia, affecting an estimated probability affecting 672 mm^3^ of the GM despite a more extensive 1464 mm^3^ of the relative decrease in the chronic samples (Liloia et al., 2021). While antipsychotics have been implicated in tissue loss (Arnone et al., 2009; Vita, De Peri, Deste, Barlati, & Sacchetti, 2015), their role in a relative increase in the GM has been less well established (Feng et al., 2020; Yue et al., 2016; Zhang et al., 2018). In a unique cohort with a very long duration of untreated schizophrenia (20 years), Liu and colleagues observed a relative increase in GM compared to duration-matched but medicated patients with chronic schizophrenia (Liu et al., 2020). More recently, Chopra et al. demonstrated antipsychotic-induced subcortical volume increase as a correlate of treatment response in first-episode schizophrenia (Chopra et al., 2021). Our report of a relatively higher GM in NN-FES adds to these findings, and clarifies that higher GM is not a late-stage phenomenon, as speculated previously (Guo et al., 2016), but occurs very early in the illness, even before antipsychotic exposure.

The most prominent lower GMC in patients with psychosis was located on the insula, inferior frontal gyrus, anterior cingulate and superior temporal gyrus, which were also observed in our VBM analysis. The two lower GMC components were distributed across four functional networks; anterior insular cortex and anterior cingulate comprising the mid-cinguloinsular salience network (Seeley et al., 2007), superior temporal gyrus comprising the primary auditory network, precuneus and inferior parietal lobule constituting the posterior aspect of the pericentral default mode network, and cuneus and lingual gyrus being part of the visual network (Uddin, Yeo, & Spreng, 2019). This result is highly consistent with the findings of previous meta-analysis studies (Chan, Di, McAlonan, & Gong, 2011; Glahn et al., 2008; Honea, Crow, Passingham, & Mackay, 2005) and a recent international mega-analysis study on antipsychotic treated patients at a clinically stable state (Gupta et al., 2015). Moreover, in a prior meta-analytical study, we observed that the GM deficits affecting insula, STG, and ACC are seen in association with expression of the schizophrenia phenotype, but not in high-risk relatives (HRR) of patients with schizophrenia (Palaniyappan, Balain, & Liddle, 2012). Taken together, these findings suggest that lower GM of these key regions exist at a drug-naive first episode stage of the illness and continue to be present despite antipsychotic treatment in later stages.

We did not observe a relationship between components with lower GM and cognition/symptom scores. This suggests that the relative reduction of GM at the time of onset is unlikely to be a major determinant of the severity of illness at presentation, though this may relate to illness persistence or non-response (Li et al., 2019). In fact, recent studies support the notion that GM reduction in schizophrenia varies with periods of active illness after the onset (Lepage et al., 2020), lifestyle factors (Moser et al., 2018) and high doses of medication exposure (Ho, Andreasen, Ziebell, Pierson, & Magnotta, 2011). Subgroups of patients with markedly lower GM do not have a consistent pattern of pronounced symptoms (Honnorat, Dong, Meisenzahl-Lechner, Koutsouleris, & Davatzikos, 2019; Planchuelo-Gomez et al., 2020) or cognitive burden in schizophrenia (Pan et al., 2020; Planchuelo-Gomez et al., 2020). In contrast, we noted that patients with a shorter duration of illness, less severe positive symptoms and better set-shifting ability had higher GM in the covariance network comprised of fronto-temporal regions and putamen. With a longer DUP (as well as age), the effect of higher GM diminished in our sample, indicating that any resilience conferred by GM increase may not be sustained over longer periods in untreated states. Given the cross-sectional nature of our data, it is not possible to make causal inferences in this regard. Longitudinal studies are required to clarify if higher GM is a marker of resilience that contributes to early help-seeking despite low levels of symptom burden.

In VBM analysis, we found both groups to show a negative correlation between GMC and age, with age-related lower GMC being much more extensive in patients (online Supplementary Fig. S6). This result was consistent with our previous finding of extensive and excessive age-related cortical thickness loss in frontal, temporal, parietal, and cingulate gyri in patients (Lin et al., 2019) and other previous studies reported faster GM loss in cortical volume in first-episode patients with schizophrenia (Arango et al., 2012; Cahn et al., 2002; Whitford et al., 2006). The age-associated GMC reduction found in neuroleptic-naïve first-episode schizophrenia provides evidence for age-related accelerated brain changes independent of antipsychotic effects.

Our study has a number of strengths that could have contributed to our ability to observe both higher and lower GM in schizophrenia. We recruited a somewhat homogenous, single-regional (Chengdu) sample whereby the variance due to referral pathways and catchment demographics were minimal. Our continuous sampling of all referrals to the hospital eliminated the bias of selecting only the most unwell subjects that are accepted to treatment programmes. Furthermore, with a higher upper bound for age of inclusion in our first-episode programme, we achieved better sex balance in the sample (Bora, Fornito, Yücel, & Pantelis, 2012) that captures the true prevalence pattern of schizophrenia in the community (Huang et al., 2019). We were able to scan participants before they were started on medications and utilised a multivariate approach that included both cortical and subcortical structures; this approach improved our sensitivity to detect putative compensatory changes compared to mass univariate methods and approaches restricted to the cortical mantle. We also had a number of limitations. We had only a single scan from our sample and no long-term outcome records, thus we were not able to relate the trajectories of illness to the brain structure at the onset. We also lacked information about lifestyle factors and other unconventional interventions that some of the patients might have received before their presentation. Finally, due to the challenging nature of the sampling procedure and the long time required for this, we used two different scanners in Chengdu. The diagnostic effects were stable across the scanners, but the increased GMC in the cerebellum was not seen in the smaller of the two datasets; we urge caution in generalising the results pertaining to this component. As in most other imaging studies, we excluded patients incapable of consenting and those who could not be scanned before instituting treatment (e.g. high degree of hostility/aggression); this may affect the generalisation of results to patients with more adverse outcomes. We focused on the GMC in the present study given its sensitivity in picking up group-level differences when comparing patients with schizophrenia and HC; covariance analysis using cortical thickness (Palaniyappan, Al-Radaideh, Gowland, & Liddle, 2020), gyrification (Das et al., 2018) and surface area (Prasad et al., 2021) are apt candidates for future studies to determine the surface level cortical contribution to regions with relatively higher GMC.

## Conclusion

In summary, we provide evidence supporting relatively higher GM occurring alongside a more extensive lower GM in early stages of schizophrenia, in association with a favourable clinical profile even before the onset of treatment. Structural cortical reorganisation in response to early deficits may occur in schizophrenia well before the onset of illness and treatment exposure. Such compensatory processes may indeed be contributing to the unique profile of this illness (late developmental onset, relapsing and remitting course with partial recovery) when compared to neurodegenerative or early developmental disorders, as well as to the heterogeneity of its clinical course.

## Supplementary material

For supplementary material accompanying this paper visit https://doi.org/10.1017/S0033291722000058. click here to view supplementary material
