# Supplementary material for: Ameliorative patterns of grey matter in patients with first-episode and treatment-naïve schizophrenia
Source: Psychol Med. 2022 Feb 15;53(8):3500–10. doi: 10.1017/S0033291722000058 (PMC10277763; doi:10.1017/S0033291722000058)
Supplement: Supplementary file 1 [file S0033291722000058sup001.docx]

### Additional details of methods:

##### 1.1 Magnetic resonance imaging (MRI) data acquisition

**Scanner 1 Dataset:** 206 patients and 169 healthy controls were scanned on a Signa 3.0-T scanner (General Electric, Medical Systems, USA) in the Department of Radiology at West China Hospital. A water phantom was scanned to get data quality assurance indexes every time, which was used to evaluate the stability of the MRI machine. High resolution T1 images were acquired by 3D spoiled gradient echo sequence (SPGR) as follows: repetition time 8.5 ms, echo time 3.93 ms, ﬂip angle 12°, slice thickness 1 mm, single shot, ﬁeld of view 24 cm*24 cm, matrix 256*256 and voxel size 0.47*0.47*1 mm3 and 156 contiguous coronal slices were collected. The quality of the brain images was examined immediately after each scans, the scans were repeated if gross distortions were found.

**Scanner 2 Dataset:** 137 patients and 172 healthy controls were scanned on a 3.0 T MR scanner (Achieva; Philips, Amsterdam, the Netherlands) using an eight-channel phased-array head coil in the department of Radiology at West China Hospital Tibet People's Government in Chengdu Office Branch. Foam padding and earplugs were used to minimize head movement and scanner noise. High-resolution T1 images were acquired by 3-D magnetization-prepared rapid gradient-echo sequence as follows: repetition time 8.37 ms, echo time 3.88 ms, flip angle 7°, in-plane matrix resolution 256 * 256, field of view 24 * 24 cm^2^, and number of slices 188. The quality of the brain images was examined immediately after each scans, the scans were repeated if gross distortions were found.

##### 1.2 T1 Image preprocessing

T1 Images were processed using Diffeomorphic Anatomical Registration Through Exponentiated Lie algebra (DARTEL) toolbox in Statistical Parametric Mapping (SPM) 8, and followed the standard approach of Ashburner (Ashburner, [2007](https://www.cambridge.org/core/journals/psychological-medicine/article/temporal-reproduction-and-its-neuroanatomical-correlates-in-adults-with-attention-deficit-hyperactivity-disorder-and-their-unaffected-firstdegree-relatives/9AF7D4EDAA163A77450E2879537ED711/core-reader#ref4)). The details are as follows: Firstly, T1-weighted images were realigned manually according to the anterior commissure-posterior commissure line and midsagittal plane. Secondly, All the 685 T1-weighted images were segmented into probability maps of gray matter (GM), white matter (WM) and cerebrospinal ﬂuid in SPM8. The resulting GM and WM probability maps were automatically rigidly aligned (3 rotations and 3 translations) to MNI space and resampled into 1 mm isotropic voxels. Thirdly, Flow fields and a series of template images were produced by running the ‘DARTEL (create templates)’ routine using imported versions of the GM and WM generated in the previous step. Forthly, the flow fields as well as the final template images were used to generate smoothed (6 mm full-width at halfmaximum isotropic Gaussian kernel), unmodulated and spatially normalized GM in Montreal Neurological Institute (MNI) space. Finally, gray matter concentrations (GMCs) were retained for subsequent statistical analysis.

##### 1.3 CANTAB Measures and procedure

Principal Component Analysis (PCA) with an orthogonal rotation (varimax) algorithm (assuming the components to be distinctive) was performed on raw indices of CANTAB including 6 subsets (Rapid Visual Information Processing, RVP; Pattern Recognition Memory, PRM; Delayed Matching to Sample, DMS; Spatial Working Memory, SWM; Intra-Extra Dimensional Set Shift, IED; Stockings of Cambridge, SOC).

 PCA was conducted in all subjects. The Kaiser–Meyer–Olken measure was used to test for sampling adequacy. Bartlett's test of sphericity was used to test if the correlation matrix was an identity matrix. The number of components to be extracted was determined based on the [eigenvalue](http://topics.sciencedirect.com/topics/page/Eigenvalues_and_eigenvectors) > 1 criterion and by examining the Scree plot (Supplementary figure 3). The ‘components’ identified were interpreted as clinically meaningful dimensions and subsequently labeled. Component scores were calculated by converting cognitive test indices to z-scores and then calculating the average of the z-scores that corresponded to each component as is done in previous studies ([Bell et al., 2009](http://www.sciencedirect.com/science/article/pii/S0920996414002503#bb0040) ;  [Mancuso et al., 2011](http://www.sciencedirect.com/science/article/pii/S0920996414002503#bb0195)). These component scores were used for further correlation analyses with SBM component loading in patients.

### 2. Dataset 1 (GE 3T scanner): Demographic and clinical characteristics and independent SBM analysis

##### 2.1 Demographic and clinical features of dataset 1

The profile of subjects studied using the GE scanner is presented in supplementary table 1. The two groups were well matched for age, gender. While NN-FES showed lower educational years than HC.

Supplementary Table 1. Demographic and Clinical characteristics of subjects on GE scanner

|  | schizophrenia  (n=206) | Control  (n=170) | df | T / χ2 value | P value |
| --- | --- | --- | --- | --- | --- |
| Age(years) | 24.12(7.26) | 23.87 (6.14) | 374 | 0.37 | 0.72 |
| Years of education | 12.05(3.07) | 13.92(2.92) | 374 | -6.00 | 0.00 |
| Gender (M/F) | 98/108 | 84/86 | 1 | 0.13 | 0.72 |
| DUP (months) | 3.00(1, 14.25)* |  |  |  |  |
| Positive | 26.94(6.26) |  |  |  |  |
| Negative | 21.00(9.16) |  |  |  |  |
| Disorganization | 33.33(6.89) |  |  |  |  |
| Excitement | 21.53(5.41) |  |  |  |  |
| Emotion | 22.53(6.28) |  |  |  |  |

*median (quarter; three quarters);

2.2 SBM Results for dataset on GE scanner

We observed 5 spatial patterns showing a significant effect of diagnosis (Supplementary fig 1). All maps in Supplementary figure 1 were thresholded at |z| > 3 and superimposed onto a standard brain template provided by MRICron. Components 3, 5, 26 and 19 contained areas where GMC values were greater in HC than NN-FES, while components 2 had areas where GMC was higher in NN-FES than HC at the P < 0.05 corrected by FDR.


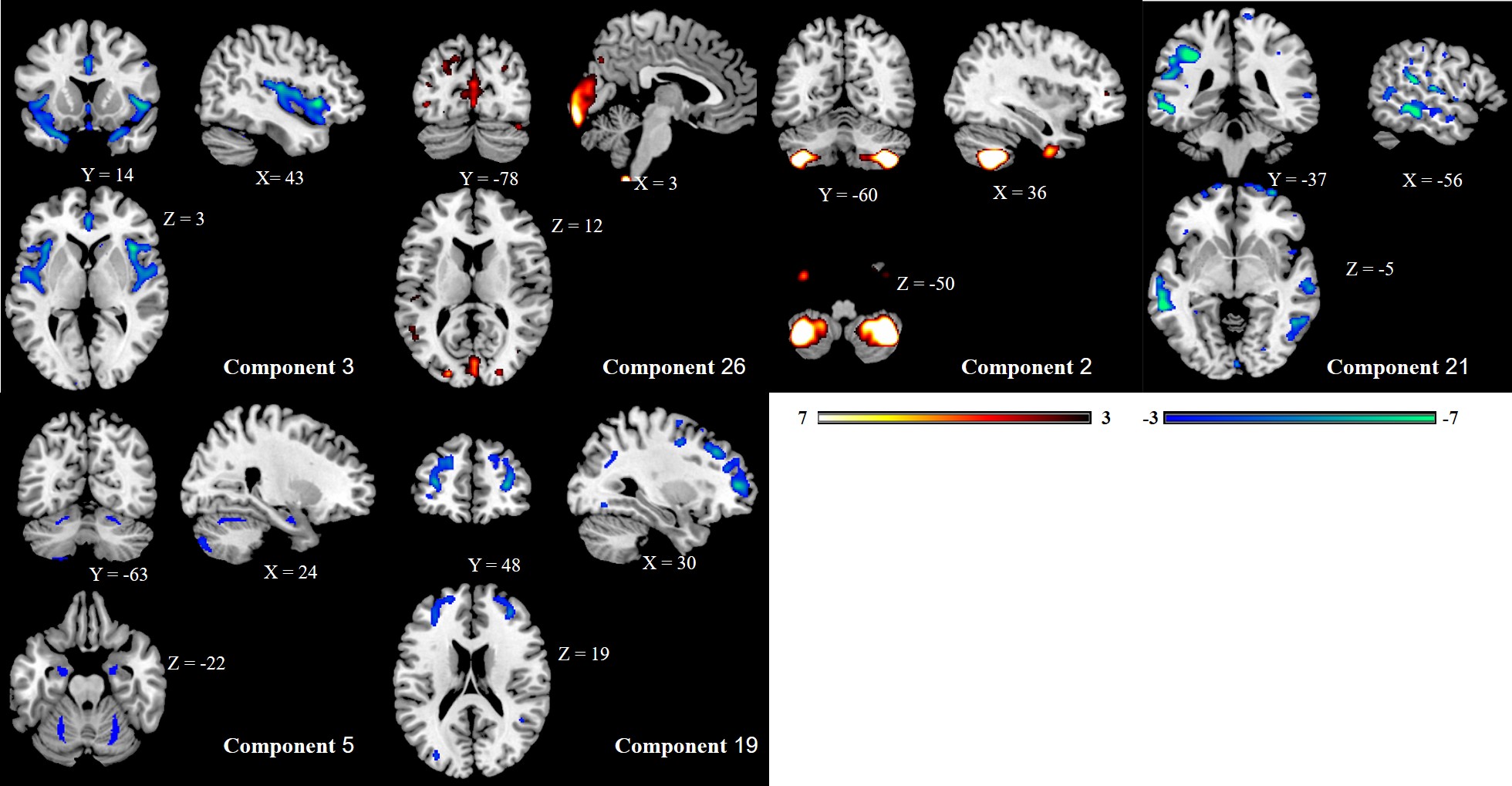


Supplementary figure 1. Spatial maps of the 5 components showing (NN-FES /HC) group effect of dataset 1 on GE scanner. All are thresholded at |z| > 3. The color bar indicates the color mapping for the normalized component weights. Components on first line (3,26,2,21) are consistent with the findings from aggregate data; 5 and 19 were components restricted to this dataset 1 (GE scanner) with no corresponding diagnostic effects in the aggregate data. HC > NN-FES: component 3, 26, 5, 19; NN-FES > HC: component 2 (at p < 0.05 FDR corrected), component 21 (p = 0.064). SBM, source-based morphometry; HC, healthy control; NN-FES, ﬁrst-episode neuroleptic-naïve patients with schizophreniform psychosis and schizophrenia. Note that the component numbers used here differ from the numbers used for the aggregated dataset in the main analysis.

### 3. Dataset 2 (Philips 3T scanner): Demographic and clinical characteristics and independent SBM analysis

##### 3.1 Demographic and clinical features of dataset 2

The profile of subjects studied using the Philips scanner is presented in supplementary table 2. The two groups were well matched for age but not for gender. NN-FES also showed lower educational years than HC.

Supplementary Table 2. Demographic and Clinical characteristics of subjects on Philips scanner

|  | schizophrenia  (n=137) | Control  (n=172) | df | T / χ2 value | P value |
| --- | --- | --- | --- | --- | --- |
| Age(years) | 23.23(6.85) | 24.80 (5.16) | 307 | -2.30 | 0.22 |
| Years of education | 11.83(2.85) | 15.55(2.85) | 307 | -11.39 | 0.00 |
| Gender (M/F) | 72/65 | 63/109 | 1 | 7.86 | 0.00 |
| DUP (months) | 6.00(2,18)* |  |  |  |  |
| Positive | 25.07(6.16) |  |  |  |  |
| Negative | 27.70(9.52) |  |  |  |  |
| Disorganization | 30.23(8.45) |  |  |  |  |
| Excitement | 22.79(7.17) |  |  |  |  |
| Emotion | 22.58(6.44) |  |  |  |  |

*median (quarter; three quarters);

##### 3.2 SBM results for dataset 2 on Philips scanner.

4 spatial patterns showed a significant effect of diagnosis (Supplementary fig 2). All maps in Supplementary figure 2 were thresholded at |z| > 3 and superimposed onto a standard brain template provided by MRICron. Components 2, 8, 1, and 7 contained areas where GMC values were greater in HC than NN-FES at the P < 0.05 corrected by FDR, while component 20 had areas where GMC was higher in NN-FES than HC at the P < 0.05 uncorrected.


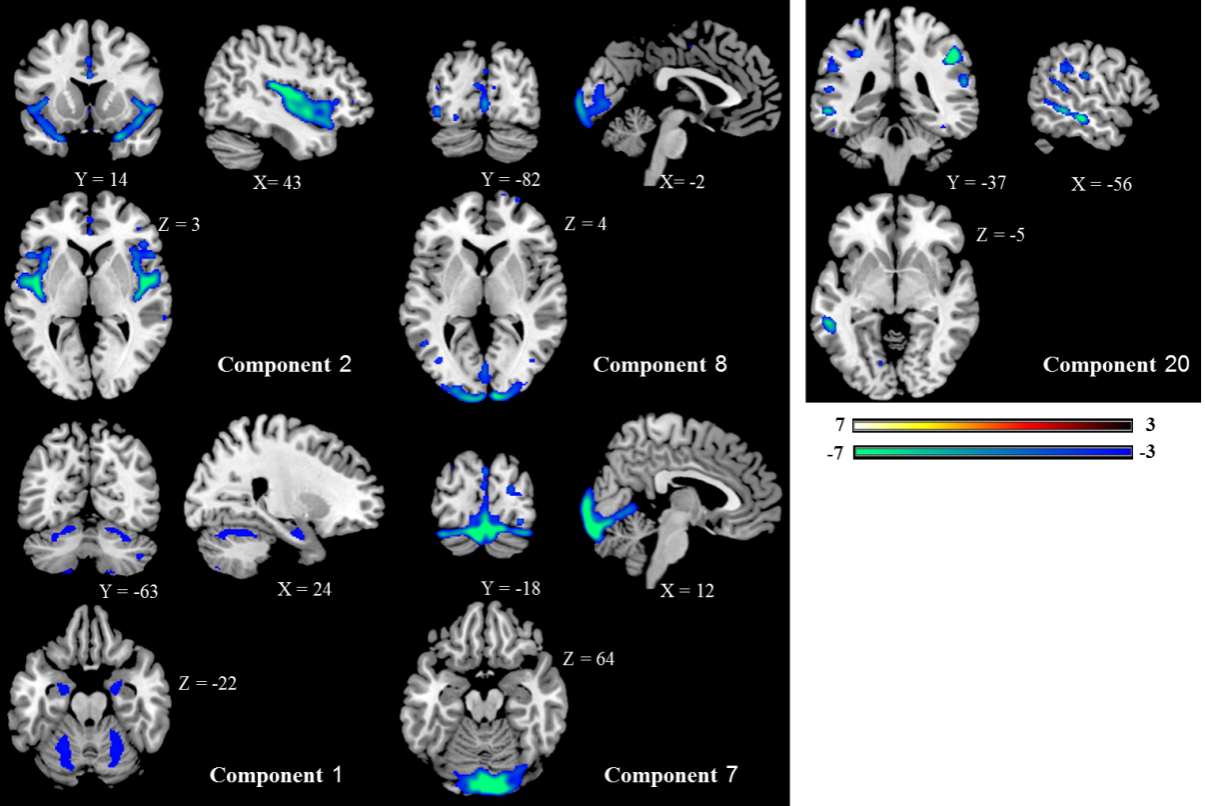


Supplementary figure 2. Spatial maps of the 4 components showing (NN-FES /HC) group effect of dataset 2 on Philips scanner. All are thresholded at |z| > 3. The color bar indicates the color mapping for the normalized component weights. Components on first line (2, 8 and 20) are consistent with findings from the aggregate data; 1 and 7 were components restricted to this dataset 2 (Phillips scanner) with no corresponding diagnostic effects in the aggregate data.. HC > NN-FES: component 2, 8, 1 and 7 (at p < 0.05 FDR corrected); NN-FES > HC: component 20 (at p < 0.05 FDR uncorrected). SBM, source-based morphometry; HC, healthy control; NN-FES, ﬁrst-episode neuroleptic-naïve patients with schizophreniform psychosis and schizophrenia. Note that the component numbers used here differ from the numbers used for the aggregated dataset in the main analysis.

### 4. The influence of scanner/site differences on SBM analysis

Calhoun’s group who first introduced the SBM approach used in this paper has considered the issue of scanner effects in depth. In their most recent review, they recommend site to be accounted for post-SBM(C. Gupta, Turner, & Calhoun, 2019; C. N. Gupta et al., 2015). In particular, they recommend a multivariate analysis of covariance (MANCOVA) model with site as a dummy-scored covariate, along with other covariates of interest(C. Gupta et al., 2019).

We took several steps to ensure scanner effects did not confound our results. Firstly, we used the same field strength (3T), as this is the most important scanner-related parameter that affects SBM (Chen et al., 2014). We also used the same acquisition protocol for both patients and controls within each site. Secondly, we pre-processed the data (i.e. registration, tissue segmentation and global volume estimation) using the same analytical pipeline. Third, we followed the recommendations of the original authors of Source Based Morphometry software (C. Gupta et al., 2019; C. N. Gupta et al., 2015) on the aggregated data and used scanner (i.e., site of origin) as a covariate when seeking diagnostic effect (Main Results). Fourth, to establish that diagnostic effects for the 4 aggregated components are present in each scanner-specific subset, we used MANCOVA (aggregate components as dependent variables, diagnosis as a factor, age, gender, educational years and intracranial volume as covariates) to test the differences between NN-FES and HC on Scanner 1 / Scanner 2, separately. The result showed a diagnostic effect for all of the four components but no other components for both datasets, confirming that the scanner differences do not explain the effects we report, and the aggregated dataset truly reflects the diagnostic effects within each subset of data.

Supplementary Table 3: MANCOVA results showing diagnostic effect when each scanner-specific samples was analyzed separately after recovering components from the aggregated dataset.

|  | **FES *VS* HC**  **on scanner 1** | | **FES *VS* HC**  **on scanner 2** | |
| --- | --- | --- | --- | --- |
| **Aggregated component numbers** | F value | P value | F value | P value |
| **Component 4** | 23.327 | <0.001 | 5.026 | 0.026 |
| **Component 13** | 6.418 | 0.012 | 4.595 | 0.033 |
| **Component 24** | 4.731 | 0.030 | 8.774 | 0.003 |
| **Component 26** | 3.971 | 0.047 | 4.286 | 0.039 |

Finally, as shown above in sections 2.2 and 3.2, we applied the ICA analysis for the dataset 1 from GE scanner and dataset 2 from Philips scanner separately, to establish if the blind source separation procedure yielded spatially similar components in the scanner-specific subsets (supplementary sections 2 and 3). Components 4, 24 were consistent in 2 datasets at the P < 0.05 corrected by FDR. Component 13 replicated only in the larger dataset from the GE scanner at the P < 0.05 corrected by FDR, with no equivalent components with diagnostic effect seen in the smaller dataset from the Phillips scanner even at liberal thresholds (0.05 to 0.1). Component 26 replicated in the Phillips scanner dataset at an uncorrected P < .05 and in the GE scanner dataset at P = 0.064. We summarise these results in Supplementary Table 4.

In summary, the 4 components recovered from the aggregated dataset from both scanners showed diagnostic effect in the same direction when patients vs. controls comparison was repeated in each dataset separately. When component selection itself was done independently in each dataset, patient vs. control comparison was consistent for 2 components of GMC reduction and to some extent, for one of the components with higher GMC in patients. As component 13 was not independently recovered in one of the datasets, we caution the readers on generalizing our findings in relation to cerebellum, on which this component loaded the most.

Supplementary Table 4 Replication of the diagnostic effect in components independently recovered from scanner-specific datasets. Numbering follows the convention used for the aggregated dataset; see supplementary figures 1 and 2 for the dataset-specific components from SBM decompositions.

| **Aggregated SBM** | Component 4 | Component 24 | Component 13 | Component 26 |
| --- | --- | --- | --- | --- |
| Dataset1 GE scanner | ++ | ++ | ++ | * |
| Dataset2 PH scanner | ++ | ++ |  | + |

The overlapping components passing FDR correction are indicated as ++ and those significant at p<0.05 uncorrected are depicted as +. * P=0.064

Supplementary Table 5: Differences in the clinical/demographic profile of patients in the two datasets

|  | FES-GE  (n=206) | FES-PH  (n=137) | df | T / χ2 value | P value |
| --- | --- | --- | --- | --- | --- |
| Age of onset | 23.06(7. 41) | 22.23 (6. 85) | 289 | 0.81 | 0.42 |
| DUP(months) | 12.56(25.50) | 14.99(21.27) | 289 | -0.79 | 0.43 |
| PANSS Positive | 26.95(6.26) | 25.07 (6.16) | 293 | 2.51 | 0.01 |
| PANSS Negative | 21.01(9.16) | 27.70(9.52) | 293 | -6.00 | <0.01 |
| PANSS Disorganization | 33.33(6.89) | 30.23(8.45) | 293 | 3.43 | <0.01 |
| CANTAB |  |  |  |  |  |
| Component 1 | -0.64(0.91) | -0.61(0.89) | 208 | -0.24 | 0.81 |
| Component 2 | 0.16(1.25) | 0.41(1.38) | 208 | -1.38 | 0.17 |
| Component 3 | 0.05 (1.08) | -.03(1.69) | 132.41 | 1.87 | 0.07 |
| Component 4 | -0.22(1.17) | -0.23(1.25) | 208 | 0.07 | 0.94 |

### 5. Results of principal component analysis of CANTAB

Four distinct and interpretable components explaining 77.15% of the variance were obtained from the PCA. Extraction communalities (amount of variance in each variable that is accounted for in the factor solution) for all variables were > 0.5 (range 0.522 to 0.867), hence none of the individual items were excluded from further analysis. The Kaiser–Meyer–Olken sampling adequacy measure was 0.774; indicating compact patterns of correlation and that the data was adequate for FA. Bartlett's test of sphericity was significant (χ^2^ = 11169.568, df = 120, p < 0.0001), indicating that the correlation matrix was not an identity matrix, i.e., there were some relationships between variables, which would yield distinct components. Variables were assigned to components based on their highest level of loading. These four components were then rotated to yield a more interpretable component structure, which converged on 5 iterations. (Supplementary table 6, Supplementary Figure 3).

Component-1 (attention, memory and planning ability) comprised of Rapid Visual Information Processing Probability of hit (RVP-PH), RVP-Latency (RVP-L), RVP-A’(sensitivity calculated using signal detection theory, the ability to discern between information-bearing patterns), Spatial Working Memory total Error (SWM-TE), SWM-strategy (SWM-Stra), Delayed Matching to Sample percent correct of all delays tests (DMS-PC-A), Pattern Recognition Memory percent correct in delay test (PRM-PCd) and Stockings of Cambridge Problems solved in minimum moves (SOC-PS). Higher score is better for component 1.

Component-2 (cognitive set shifting ability) was comprised of IED Stages completed (IED-SC, the number of trials undertaken on all successfully completed stages), IED Total trials (IED-TT, the number of trials completed on all attempted stages with an adjustment for any stages not reached) and IED Total errors (IED-TE, a substantial number of errors, a measure of one’s efficiency in attempting the test). The lower score of component 2, the better cognitive set shifting ability.

Component-3 (response bias using signal detection theory, the ability to discern between information-bearing patterns) was comprised of RVP Probability of false alarm (RVP-PFA) and RVP B″(measure the tendency to respond regardless of whether the target sequence is present). Higher is better.

Component-4 (immediate memory) was comprised of DMS percent correct (DMS-PC, a correct selection was made on the subject’s first response), DMS percent correct in n ms delay (DMS-PC-S, the number of occasions upon which the subject selected the correct stimulus in trials which match the delay set by the Delay option), Pattern Recognition Memory percent correct in immediate test (PRM-PCi). Higher is better.

Supplementary Table 6 Rotated component matrix of neurocognition in all subjects (n = 463)

|  | Component 1 | Component 2 | Component 3 | Component 4 |
| --- | --- | --- | --- | --- |
| Variance explained | 44.29% | 13.65% | 9.27% | 6.52% |
| Eigenvalue | 7.09 | 2.19 | 1.48 | 1.04 |
| RVP-PH | **.903** | -.092 | .009 | .040 |
| RVP-A-PR | **.850** | -.084 | .313 | .084 |
| SWM-TE | **-.666** | .303 | -.126 | -.281 |
| SWM-Stra | **-.659** | .260 | -.073 | -.119 |
| DMS-PC-A | **.619** | -.243 | .116 | .584 |
| PRM-PCd | **.546** | -.052 | .267 | .200 |
| RVP-ML | **-.538** | .123 | -.504 | -.207 |
| SOC-PS | **.526** | -.201 | .118 | .300 |
| IED-SC | .146 | **-.965** | .060 | .097 |
| IED-TE-A | -.219 | **.957** | -.076 | -.134 |
| IED-TT-A | -.226 | **.953** | -.080 | -.136 |
| RVP-PFA | -.192 | .084 | **-.937** | -.123 |
| RVP-B-DP | .146 | -.057 | **.936** | .133 |
| DMS-PC-S | .029 | -.068 | .112 | **.825** |
| DMS-PC | .581 | -.236 | .127 | **.676** |
| PRM-PCi | .350 | -.098 | .169 | **.484** |


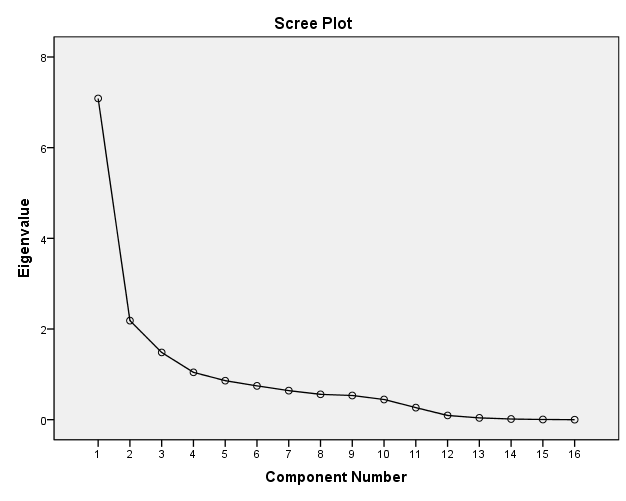


Supplementary figure 3. Scree plot of CANTAB components

Supplementary table 7. Correlation between components showing GM alteration

|  | Component 4 | Component 24 | Component 13 | Component 26 |
| --- | --- | --- | --- | --- |
| Component 4 |  | -0.441  (<0.0001) | 0.200  (0.00019) | -0.219  (<0.0001) |
| Component 24 |  |  | -0.208  (<0.0001) | 0.058  (0.284) |
| Component 13 |  |  |  | -0.099  (0.067) |

Note: R (uncorrected p value). For component 4 was a predominantly positive spatial component but components 24, 13 and 26 were predominantly negative spatial components, these correlations indicated two grey matter decreased component 4 and 24 were highly positively correlated to each other and negatively correlated to grey matter increased component 13, grey matter increased component 26 was positively correlated to component 4.


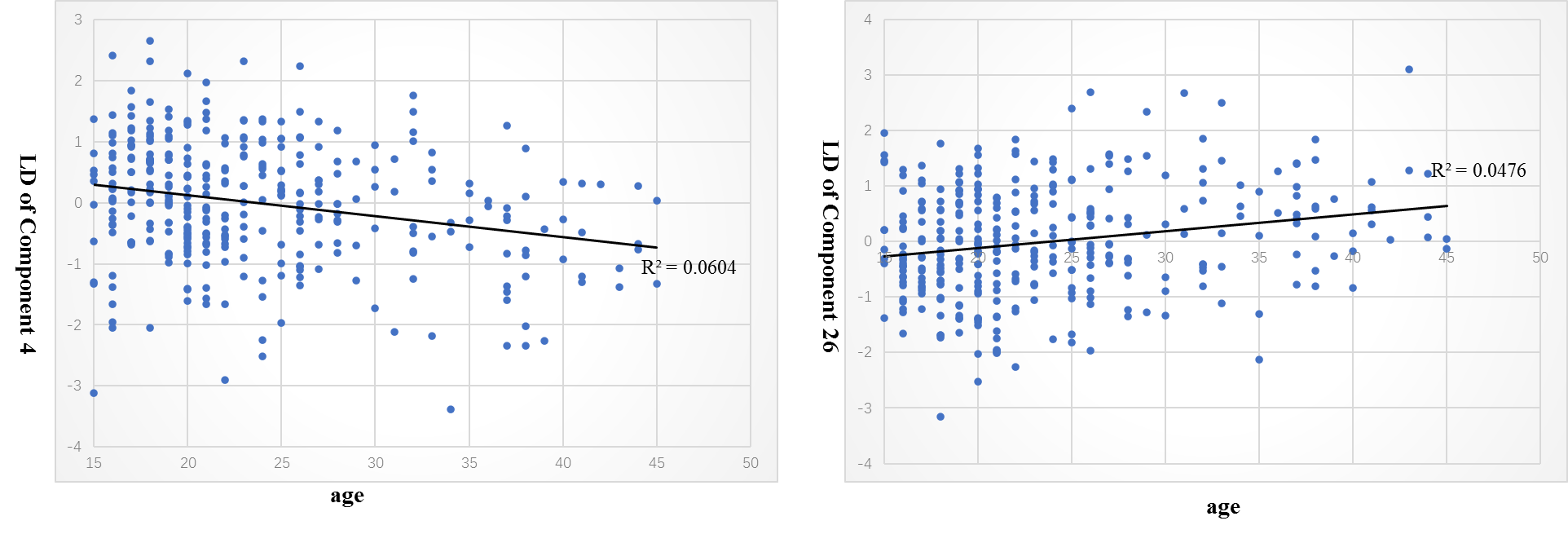


Supplementary figure 4. Component 26 as well as Component 4 were related to age in patients’ group. For component 4 is predominantly positive, component 26 is predominantly negative, this indicated component 4 and 26 were both negatively correlated to age.

LD: loading coefficient (To exclude the effects of scanner, gender and educational years on loading parameters of components, multiple regression analyses were performed on each SBM component as dependent variables, and scanner, gender, educational years and intracranial volume as independent variables, in the HC and NN-FES separately)

### 6. VBM Analysis and Results

We also performed VBM analysis on the aggregated dataset using the SPM12 software. Two sample t-tests were performed on the smoothed GMC images with scanner, age, gender, educational years and intracranial volume as covariates. A significant difference was set as the threshold of family error wise (FWE) corrected type-1 error rate of 5% and an extent threshold of 50 voxels. We also lowered the threshold at p<0.001 uncorrected at voxel level with FWE-corrected P<0.05 at cluster level to detect GM increases that are expected to be subtler and of lower effect size than GM decreases.

For clinical and neurocognitive characteristics, we used regression models in SPM12, to independently predict GMC (smoothed GMC images regressed for the covariates of scanner, age, gender, educational years and intracranial volume), with PANSS positive, negative, disorganization scores (in 295 patients) and 2 cognitive factors from PCA of CANTAB scores (in 210 patients) as independent predictors in patients with NN-FES. In a separate analysis, age was also used to predict the GMC (after adjusting for scanner, gender, educational years and intracranial volume as covariates) in the patient group and HC group separately. A significant difference was set as the threshold of family error wise (FWE) corrected type-1 error rate of 5% and an extent threshold of 50 voxels.

Compared with HC, patients with NN-FES had lower GMC in bilateral cingulate gyrus, insular, right superior temporal gyrus, medial orbital frontal, rectus, caudate, precentral gyrus and lingual gyrus at FWE-corrected P < .05 and an extent threshold of 50 voxels, no significant higher GMC was found in NN-FES group at this statistical threshold (Supplementary figure 5A). However, when we used lower threshold, higher GMC were found in caudate, cerebellar, temporal pole in NN-FES group at p<0.001 at voxel level with FEW-corrected P < 0.05 at cluster level, which overlapped with the results of greater GMC in patients reported in the SBM analysis (Supplementary figure 5B).

The VBM analysis of positive, negative, disorganized PANSS scores or 2 cognitive factors from PCA of CANTAB scores or DUP with GMC showed no significant correlation.

Both patient group and HC group showed GMC was negatively correlated to age at the threshold of FWE-corrected P < .05 and an extent threshold of 50 voxels, while patients showing much more extensive negative associations located in the frontal cortex, superior temporal gyrus, insular, parietal cortex, cingulate gyrus and cerebellum (Supplementary figure 6).


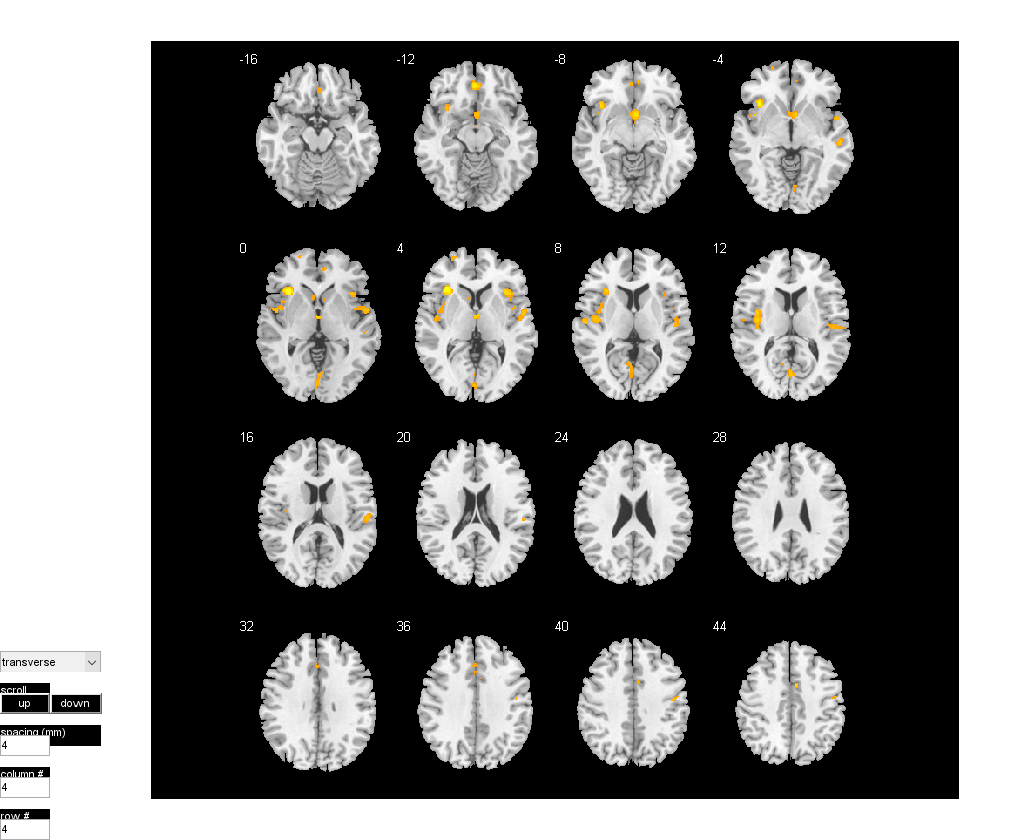

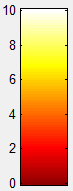
 A


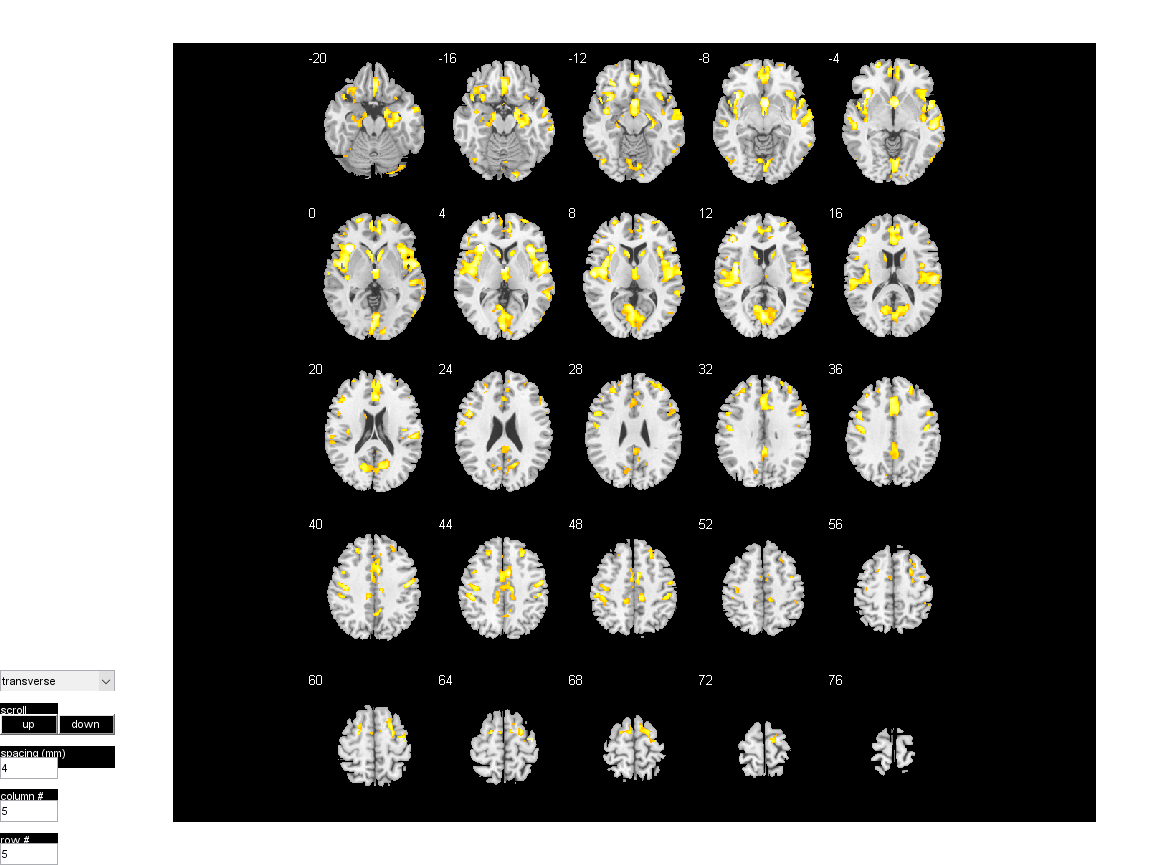

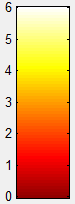

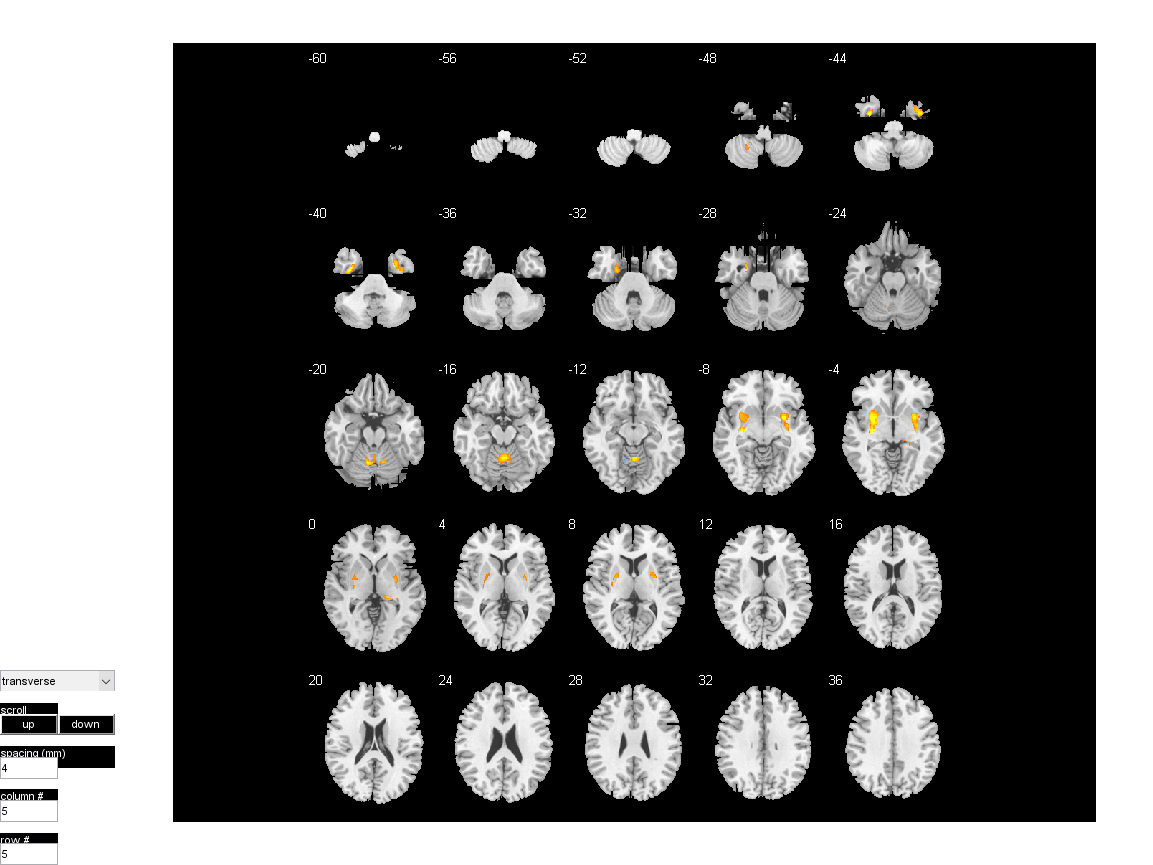
 B

Supplementary Figure 5. Results of the VBM analysis.

1. Patients with NN-FES had lower GMC in bilateral cingulate gyrus, medial orbital frontal, rectus, caudate, insular, precentral gyrus, lingual gyrus and right superior temporal gyrus at FWE-corrected P < .05 and an extent threshold of 50 voxels. No significant higher GMC was found in NN-FES group at this threshold.
2. The differences GMC between NN-FES and HC at P <0.001 at voxel level with FWE-corrected P < .05 at cluster level. Left: HC>NN-FES (i.e. decreased GMC in patients); Right: NN-FES>HC (i.e. increased GMC in patients)

Abbreviations: VBM, voxel-based morphometry; GMC, gray matter concentration; NN-FES, ﬁrst-episode neuroleptic-naïve patients with schizophreniform psychosis and schizophrenia.


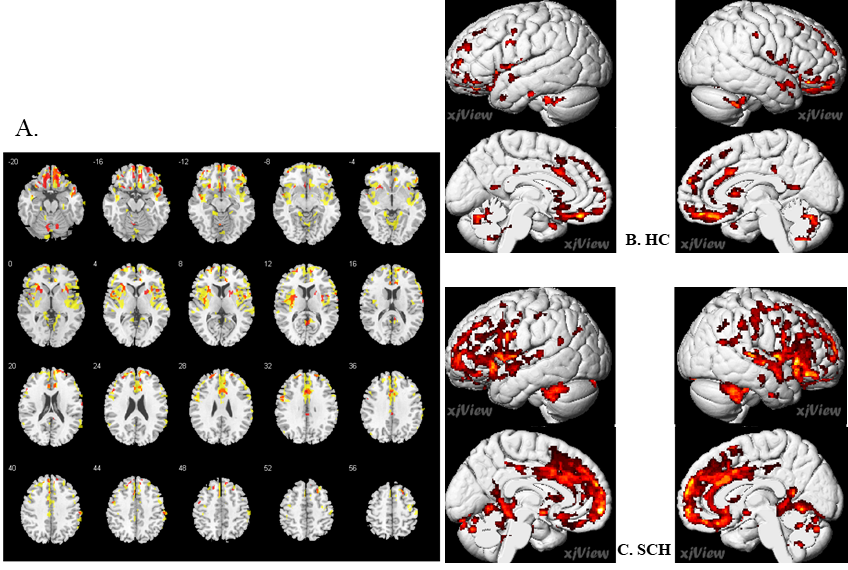


Supplementary figure 6. In VBM analysis, GMC was found negatively correlated with age at the at the threshold of FWE-corrected P < .05 and an extent threshold of 50 voxels. A. Yellow maps refer to the locations with negative age effect for the patient group; red maps refer to the healthy control group, with notable overlap. B. GMC was negative correlated with age in healthy control (HC). C. GMC was negative correlated with age in ﬁrst-episode neuroleptic-naïve patients with schizophreniform psychosis and schizophrenia (SCH).

### 7. Artifactual components and components with no diagnostic effect in the aggregated dataset


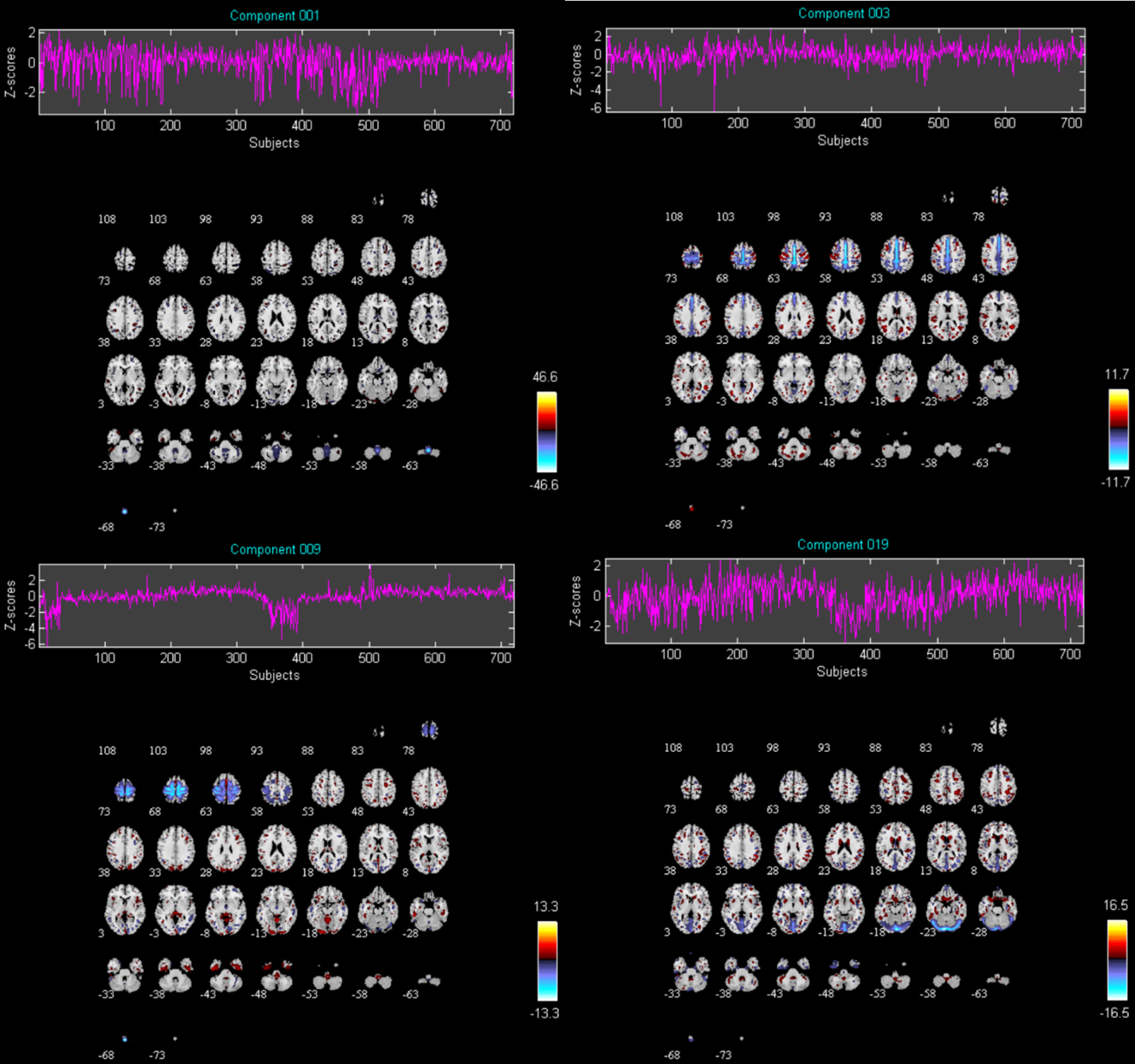


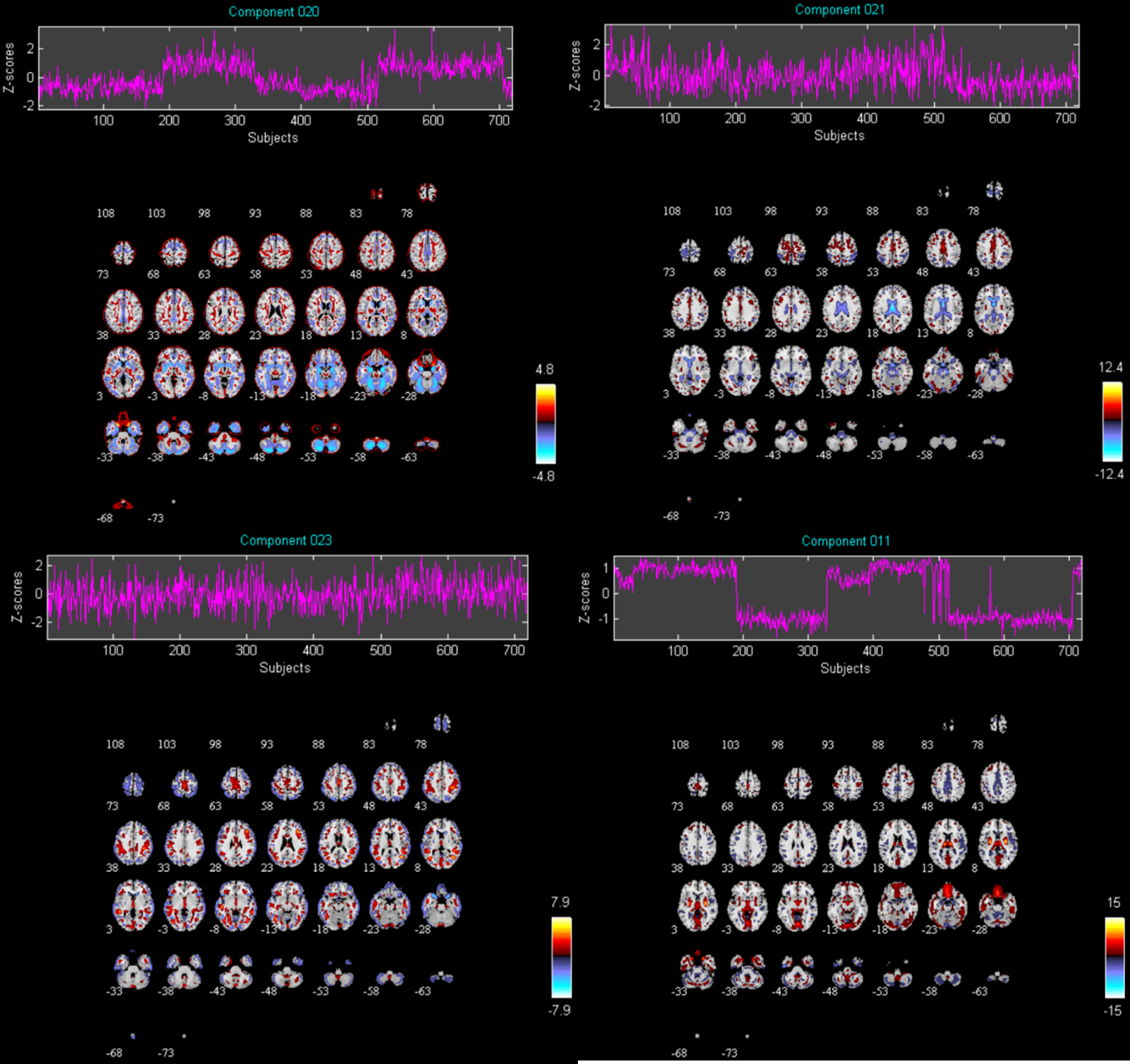


Supplementary figure 7 The discarded 8 components suggestive of obvious artifacts, i.e., having spatial patterns primarily around the edges of the brain, in white matter regions and ventricle.


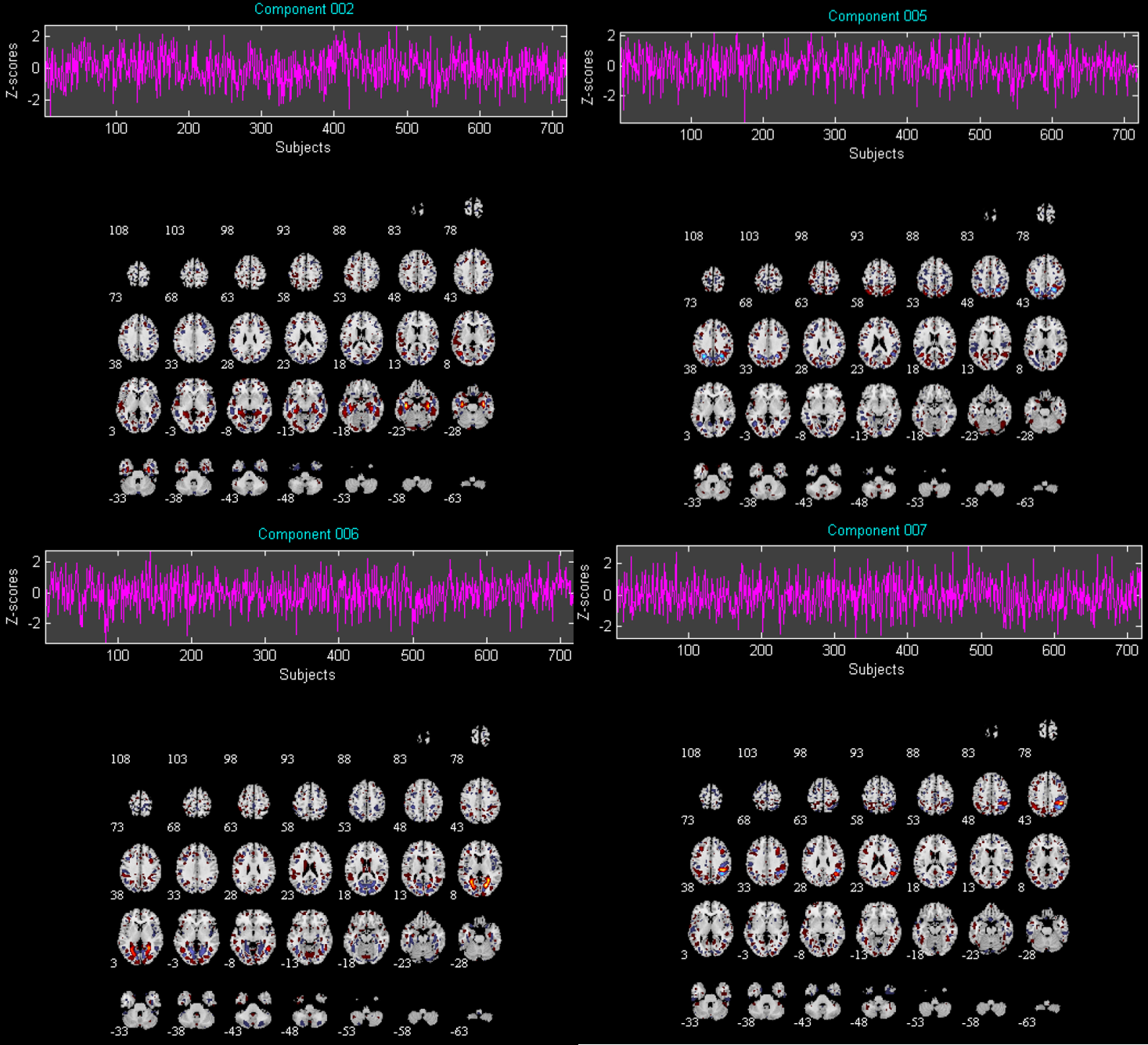


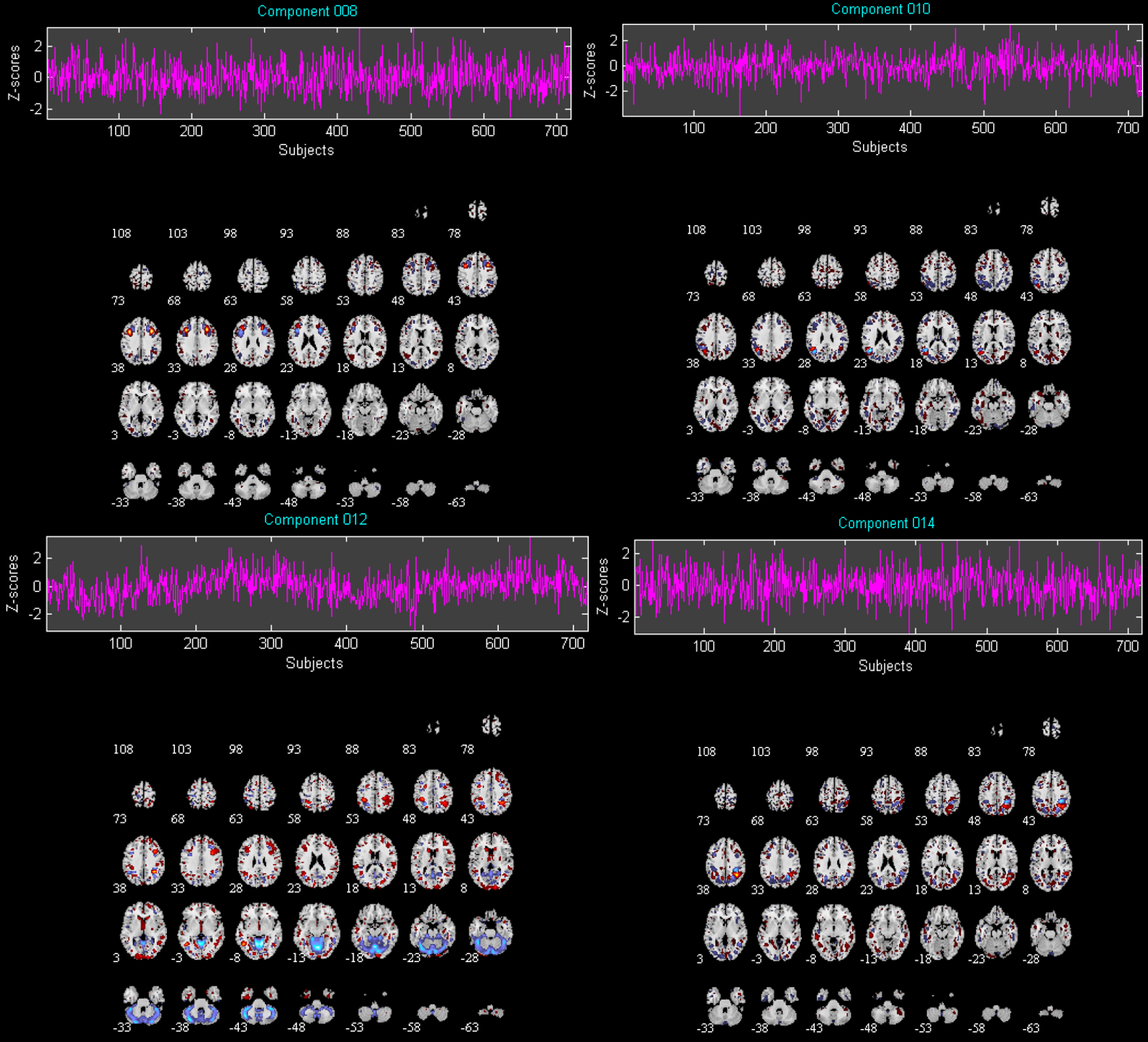


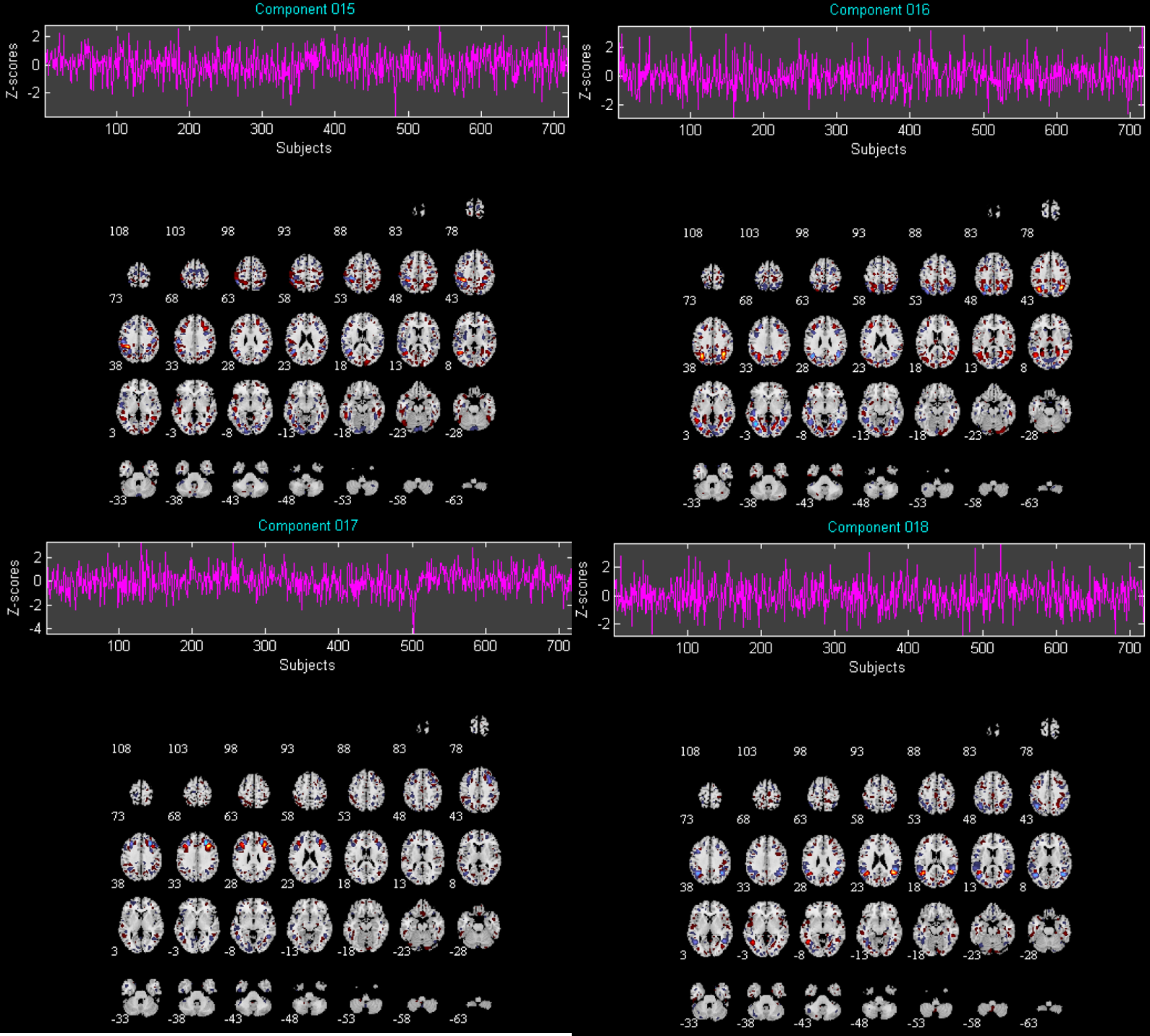


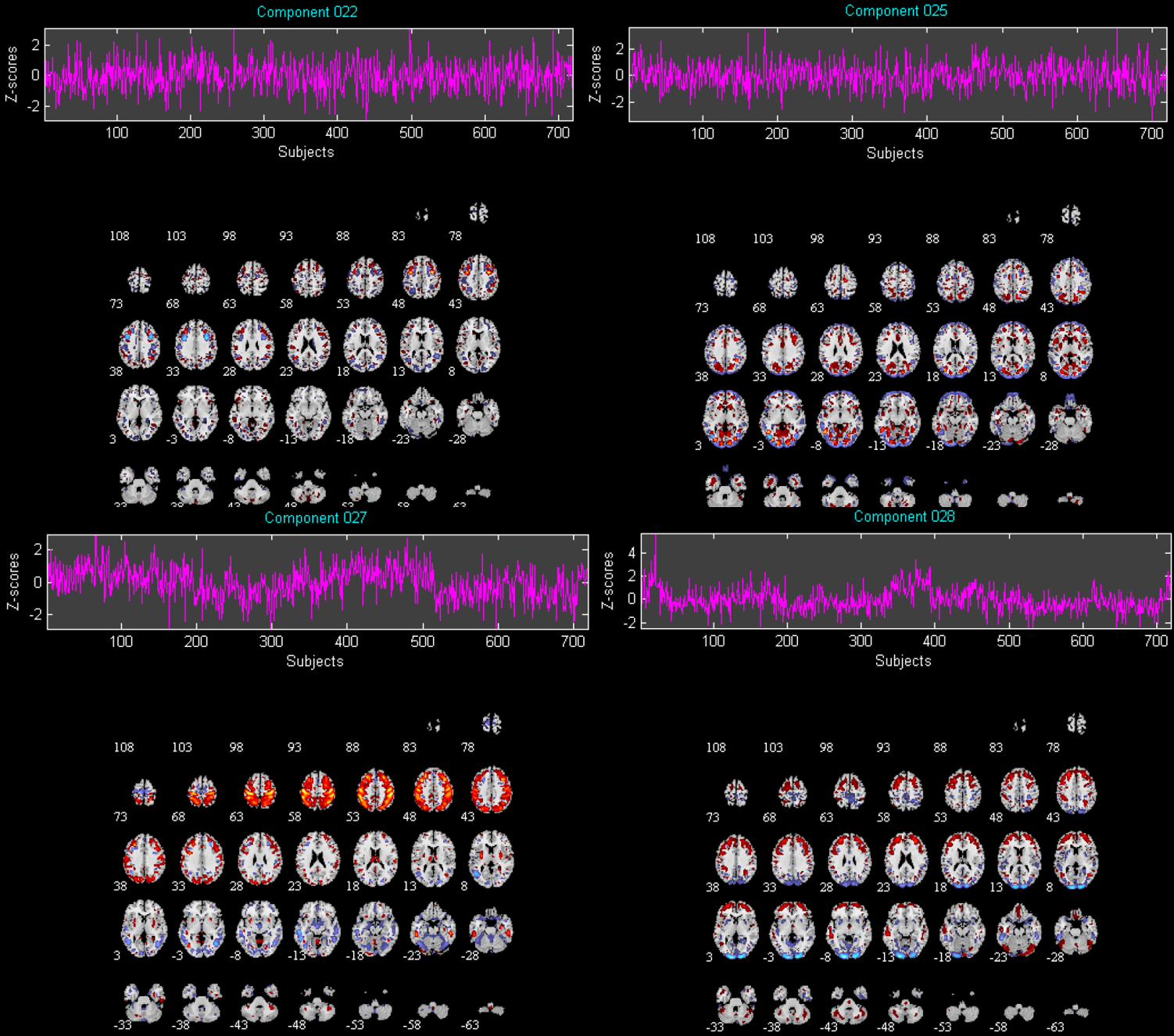


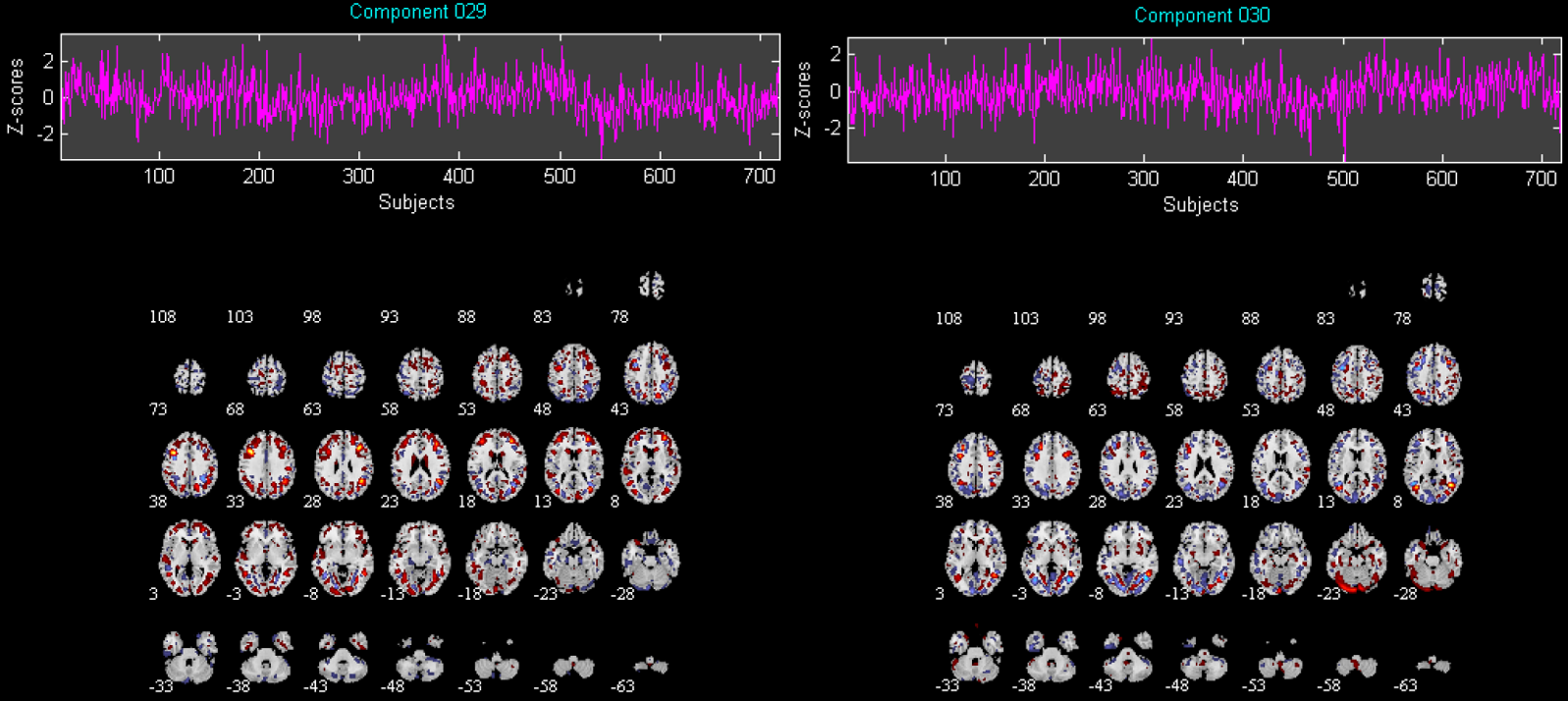


Supplementary Figure 8: The 18 components that showed no differences between patients and healthy controls.

**Refences:**

Chen, J., Liu, J., Calhoun, V., Arias-Vasquez, A., Zwiers, M., Gupta, C., . . . Turner, J. (2014). Exploration of scanning effects in multi-site structural MRI studies.*Journal of Neuroscience Methods, 230*, 37-50. doi: 10.1016/j.jneumeth.2014.04.023

Gupta, C., Turner, J., & Calhoun, V. (2019). Source-based morphometry: a decade of covarying structural brain patterns. *Brain Structure & Function, 224*(9), 3031-3044. doi: 10.1007/s00429-019-01969-8

Gupta, C. N., Calhoun, V. D., Rachakonda, S., Chen, J., Patel, V., Liu, J., . . . Turner, J. A. (2015). Patterns of Gray Matter Abnormalities in Schizophrenia Based on an International Mega-analysis. *Schizophrenia Bulletin, 41*(5), 1133-1142. doi: 10.1093/schbul/sbu177
